# Misconceptions and Lack of Knowledge of Self-Regulation of Learning Hinder Students’ Use of Self-Regulation Strategies and Their Achievement: How This Can Be Changed by a Model-Based Instructional Video

**DOI:** 10.3390/bs16040612

**Published:** 2026-04-20

**Authors:** Antonia Fischer, Charlotte Christine Dignath

**Affiliations:** Institute of Psychology, Department of Educational Psychology, Goethe University Frankfurt, 60323 Frankfurt am Main, Germany

**Keywords:** self-regulation of learning, misconceptions, conceptual change, metacognitive strategy use

## Abstract

Many students rarely use self-regulated learning (SRL) strategies, and little is known about what drives this variation. This study investigates which facets of SRL competence predict students’ reported strategy use and performance, and whether these facets can be enhanced through video-based modeling. Based on conceptual change theory, we hypothesize that learners’ initial SRL competence influences their ability to acquire SRL strategies. A total of 157 university students participated in a quasi-experimental lab study using a pre-, post-, and follow-up design. Participants watched one of three SRL modeling videos (mastery, coping, control) and completed questionnaires and reflection tasks assessing SRL beliefs, knowledge, and strategy use. Inconsistent beliefs and the interaction between self-efficacy and utility beliefs negatively predicted reported SRL use, while performance was positively associated with SRL knowledge, self-efficacy, and reported strategy use. Participants in the intervention condition showed significantly greater increases in SRL knowledge and reduced inconsistent beliefs. Most notably, learners with an initially low to average SRL strategy use showed the largest improvements following the intervention. These findings underscore the potential importance of addressing both cognitive and belief-related components of SRL. The findings suggest that modeling videos may support conceptual change and the development of SRL competence, particularly among less experienced learners.

## 1. Introduction

This article aims to identify which specific facets of learners’ SRL competence predict their use of SRL strategies. Secondly, we test how these facets can be improved. Based on conceptual change theory (Vosniadou et al., 2008), we further assume that learners’ prior SRL competence influences the acquisition of SRL strategies. A third aim of this study is therefore to test which facets of SRL competence moderate the learning of SRL.

### 1.1. Models of SRL and SRL Competence

SRL means that learners plan, monitor, and reflect on their learning process (Zimmerman, 2000). It involves cognitive, metacognitive, and motivational strategies to support their learning (Boekaerts, 1999), with metacognition playing a crucial role (e.g., Corno, 1986).

According to Winne and Hadwin’s (1998) COPES model, self-regulated learning can be understood as a cyclical process in which learners regulate their learning through five components: Conditions, Operations, Products, Evaluations, and Standards. Conditions refer to internal and external factors influencing learning (e.g., prior knowledge or task characteristics), while Operations describe cognitive processes such as searching, monitoring, and elaborating information. These processes lead to Products (e.g., notes or solutions), which learners evaluate against Standards such as goals. Based on these evaluations, learners adjust their strategies in subsequent learning cycles.

Thus, the use of SRL strategies in a specific learning situation depends on the task and cognitive conditions (Winne & Hadwin, 1998), including beliefs, motivational orientations, and strategy knowledge. Following Weinert (2001), we refer to these as facets of learners’ competence.

We further draw on Winne and Marx’s (1987) AEIOU model as well as the expectancy–value theory (Wigfield & Eccles, 2000), who state that motivational orientations include (among others) learners’ self-efficacy beliefs to be able to use SRL strategies as well as their utility beliefs regarding their usefulness for learning. The AEIOU model (Winne & Marx, 1987) emphasizes the interaction between learner characteristics and the learning environment. Together, these models show that SRL strategy use depends on knowledge of strategies, beliefs, motivations, and interpretations of learning situations. In this study, SRL competence comprises beliefs (consistent or inconsistent with SRL theory), (2a) self-efficacy to use SRL and (2b) utility beliefs about SRL strategy use, and (3) strategy knowledge. Based on the COPES model, we assume that learners’ SRL strategy use depends on these facets.

#### 1.1.1. SRL Strategy Knowledge and Its Relation to SRL Strategy Use

SRL strategy knowledge plays a key role in how learners approach tasks. Learners with greater strategy knowledge have more resources available for strategy use (Boekaerts, 1997). Empirical findings largely support this assumption. Strategy knowledge predicts strategy use in university students (Karlen & Compagnoni, 2017) and school students (Artelt & Schneider, 2015). It also predicts SRL use indirectly via self-efficacy (Cerezo et al., 2019).

However, the findings are mixed. Foerst et al. (2017) found no relation between strategy knowledge and study behavior, suggesting that knowledge does not automatically translate into use. Overall, strategy knowledge is important but not sufficient for SRL strategy deployment. Consistent with this, a meta-analysis by Simonsmeier et al. (2022) showed that prior knowledge has variable effects on learning outcomes. This suggests that additional facets of SRL competence must be considered.

#### 1.1.2. Beliefs Consistent and Inconsistent with SRL Theory and Their Relation to SRL Strategy Use

In addition to knowledge, beliefs can influence behavior (Bandura, 1991). Students’ beliefs about SRL, including task-related beliefs and values, may affect their implementation of SRL strategies (Boekaerts, 1996). However, research on learners’ SRL beliefs remains limited (e.g., Lawson et al., 2019)

Beliefs can be consistent or inconsistent with SRL theory (Vosniadou et al., 2021). Consistent beliefs (e.g., linking new to prior knowledge) positively predict strategy use, whereas inconsistent beliefs (e.g., learning should be immediate) negatively predict strategy use. Thus, misconceptions about SRL may hinder strategy deployment.

#### 1.1.3. Self-Efficacy Beliefs to Use SRL Strategies and Their Relation to SRL Strategy Use

Even with knowledge and appropriate beliefs, learners may not apply strategies if they lack confidence. Self-efficacy is therefore a key predictor of SRL strategy use. Prior research shows consistent links between self-efficacy and strategy use (e.g., Lim & Yeo, 2021). Self-efficacy predicts metacognitive strategy use (Bai & Guo, 2021) and overall SRL behavior (Li & Zheng, 2018). Studies focusing specifically on SRL-related self-efficacy also show positive effects on strategy use (Cerezo et al., 2019; Joo et al., 2000). Overall, self-efficacy appears to be a robust predictor of SRL strategy use.

#### 1.1.4. Utility Beliefs About SRL Strategies and Their Relation to SRL Strategy Use

According to expectancy–value theory (Wigfield & Eccles, 2000), learners must both expect success (self-efficacy) and value the task (utility). Thus, utility beliefs should also influence SRL strategy use. Empirical findings are mixed. Some studies report positive effects of utility beliefs (Karabenick et al., 2021), while others find no direct effect (Cerezo et al., 2019). Utility beliefs alone do not fully explain strategy use.

Instead, SRL strategy use likely depends on the interaction of multiple competence facets.

### 1.2. How Facets of SRL Competence and Strategy Use Predict Student Performance

Research combining these facets of SRL competence is rare. For example, Cerezo et al. (2019) examined how learners’ self-reported knowledge, self-efficacy and usefulness beliefs influence their SRL strategy use, but have not taken into account learners’ beliefs about SRL. The authors find that SRL training for university students led to an improvement in SRL strategy knowledge, which in turn was associated with an improvement in students’ SRL self-efficacy. Both SRL strategy knowledge and SRL self-efficacy were positively associated with students’ use of SRL strategies. Yet, students’ beliefs about the usefulness of SRL strategies were neither associated with SRL strategy knowledge, nor was the use of SRL strategies associated with SRL self-efficacy. To our knowledge, there are no further studies looking into multiple competence facets with regards to SRL strategy use.

The link between SRL and student performance is well-established (e.g., Broadbent & Poon, 2015; Dent & Koenka, 2015). However, research on which specific facet of SRL competence predicts student performance is less common. While SRL strategy knowledge has been linked to student performance (e.g., Artelt & Schneider, 2015; Chytrý et al., 2020; Soodla et al., 2017; Swanson, 1990; Tian et al., 2018), there is limited evidence examining other facets of SRL competence in relation to performance. Trentepohl et al. (2023) found that strategy knowledge about resource-management strategies indirectly affected university students’ performance via their use of such strategies. Karlen et al. (2021) also found a positive prediction effect of strategy knowledge on students’ academic achievement. Regarding beliefs of SRL, Vosniadou et al. (2021) showed that beliefs consistent with SRL positively predicted strategy use, while beliefs inconsistent with SRL theory negatively predicted strategy use. Strategy use, in turn, positively predicted university students’ academic performance. Several studies found that students’ self-efficacy for SRL predicted their academic achievement (Klassen, 2010; Schukajlow et al., 2022; Caprara et al., 2008; Zimmerman, 2013; Zuffianò et al., 2013). Kitsantas and Zimmerman (2008) discovered a prediction effect of students’ self-efficacy for learning on their later academic grades. Zimmerman et al. (1992) found a significant effect of SRL self-efficacy beliefs on students’ academic achievement via their self-efficacy for academic achievement and academic goal setting. Karlen et al. (2021) found that a student’s SRL self-concept, a concept closely related to SRL self-efficacy, positively predicts their academic achievement. Regarding utility beliefs, Schmidt et al. (2012) used prompts to encourage student reflections about the utility of the learning content, which enhanced learning motivation and comprehension. To our knowledge, there are no studies integrating all these facets of SRL competence to model student performance.

### 1.3. Conceptual Change in SRL

Learning often requires revising prior knowledge. When new information conflicts with existing beliefs, conceptual change occurs (Vosniadou et al., 2008). Prior misconceptions can hinder learning.

We assume that this applies to SRL. Learners’ misconceptions about SRL may impede both strategy use and the acquisition of new SRL knowledge.

Research on conceptual change in SRL is limited. Vosniadou et al. (2020) distinguish between beliefs consistent and inconsistent with SRL theory and emphasize that both can coexist. Addressing misconceptions is therefore essential for conceptual change.

### 1.4. Theory of Change: Example-Based Learning Using Video-Based Modeling Examples

In educational research, example-based learning is a common instructional principle, typically categorized into worked examples and modeling examples (Van Gog & Rummel, 2010). In this study, we use modeling examples to support learners’ conceptual change. Drawing on social–cognitive theory, which posits that humans learn by observing others (Bandura, 1986), modeling examples typically show a model performing a more or less complex task (Van Gog & Rummel, 2010).

In addition, modeling examples allow the model to explain their reasoning and underlying thought processes. Unlike worked examples, they are better suited for training less structured skills such as SRL, inquiry learning (Omarchevska et al., 2022), scientific reasoning (Kant et al., 2017), gymnastics skills and knowledge (Trabelsi et al., 2022), or writing (Zimmerman & Kitsantas, 2002).

#### 1.4.1. Mastery vs. Coping Modeling Examples

Modeling examples can be categorized into mastery and coping models (Van Gog & Rummel, 2010). Mastery models typically feature experts demonstrating faultless task performance (Huang, 2016), whereas coping models show more natural behavior, including errors and their correction (Van Gog & Rummel, 2010), often accompanied by verbalized doubts or low self-efficacy (Huang, 2016).

While mastery models align with Bandura’s (1986) theory of observational learning, coping models additionally emphasize learning from errors. Errors can provoke reflection and thereby deepen understanding (VanLehn, 1999). Analyzing errors and considering alternative solutions may also induce cognitive conflict (Kramarski & Zoldan, 2008), which can support conceptual change (Limón, 2001).

Both modeling types may facilitate conceptual change in SRL competence. However, coping models may be particularly effective because they first activate learners’ misconceptions and then refute them through corrected behavior. This sequence resembles refutation processes described in conceptual change research (e.g., Eitel et al., 2021).

While mastery modeling is widely studied, coping models are rarely examined independently and are typically compared with mastery models (see Van Gog & Rummel, 2010 for an overview). Studies either combine both models within one condition (e.g., Baldwin, 1992) or compare them across conditions (e.g., Huang, 2016; Schunk & Hanson, 1985; Zimmerman & Kitsantas, 2002). Findings are mixed and appear to depend on the outcome measures used (e.g., Baldwin, 1992; Cumming & Ramsey, 2011; Huang, 2016; Schunk et al., 1987). This variability may reflect interactions with instructional features, learner characteristics, or contextual factors (e.g., Min, 2016).

#### 1.4.2. Modeling Examples in the Context of SRL

Modeling examples have been successfully used to teach self-regulatory skills (see Van Gog & Rummel, 2010), including self-assessment (Van Gog et al., 2010; Wijnia & Baars, 2021), problem-solving (Raaijmakers et al., 2018; Wijnia & Baars, 2021), and task selection (Raaijmakers et al., 2018; Van Gog et al., 2010). Omarchevska et al. (2022) found that participants who watched short modeling videos used more metacognitive strategies (planning and monitoring) than those in a control condition.

Zimmerman and Kitsantas (2002) compared mastery and coping models and found that participants in the coping condition outperformed those in the mastery condition on several SRL measures, with both groups outperforming the control condition.

### 1.5. Prior Facets of SRL Competence That Might Serve as Moderators of Conceptual Change

In addition to the type of modeling (mastery vs. coping), there are specific variables that influence the learning outcomes of modeling examples. Some learner characteristics that may influence which model is most useful for learners include their prior knowledge and self-efficacy (Van Gog & Rummel, 2010).

#### 1.5.1. Prior Knowledge of SRL

Drawing on the expertise reversal effect (Kalyuga et al., 2003), learning formats that are useful when students have little prior knowledge may be less effective for students with more prior knowledge, and vice versa. Accordingly, weak learners tend to benefit more from weak models and better learners tend to learn more from good models (Van Gog & Rummel, 2010). Hence, students with little prior SRL knowledge are likely to learn better from coping models, whereas learners with an extensive SRL knowledge may benefit more from mastery models.

#### 1.5.2. Self-Efficacy Beliefs

Self-efficacy is another learner characteristic that might influence which example-based model is most useful for individual learners (Van Gog & Rummel, 2010). Schunk and Zimmerman (2007) suggest that observing a proficient model performing a task can enhance learners’ self-efficacy by fostering the belief that they too can succeed at the task. The belief to have acquired a skill by observing a model can also lead to more self-efficacy (Schunk, 1984). The more similar a model is to the observer, the more likely learners are to think they could perform the observed action themselves. Thus, coping models are likely to boost self-efficacy more than mastery models, as students acquiring a new skill are not experts in the area to be learned.

### 1.6. Self-Regulated Learning Processes and Their Assessment

Measuring SRL strategy use is challenging (Veenman & van Cleef, 2019). Numerous studies have shown that learners of all ages struggle to accurately assess their strategy use in retrospect (Roth et al., 2016). SRL questionnaires correlate only slightly to moderately with learning success and are not a valid measure of SRL (Veenman et al., 2006; Winne & Perry, 2000). Instead, it has been shown that SRL processes are situational, varying with different contexts and fluctuating intra-individually over time (Boekaerts, 1997; Hadwin et al., 2001; Theobald et al., 2019). Therefore, the measurement of SRL has to be situational. Recently, innovative methods have been developed to validly assess SRL (e.g., Azevedo et al., 2022; Winne et al., 2019).

### 1.7. The Present Study

Taken together, the present study adopts an integrative perspective on SRL competence. We conceptualize SRL competence as a multidimensional construct comprising learners’ knowledge, beliefs, and motivational orientations, drawing on Winne and Hadwin’s COPES model. Expectancy–value theory explains how self-efficacy and perceived utility shape learners’ willingness to deploy strategies, while conceptual change theory explains how misconceptions about SRL may hinder the integration of new strategy knowledge. Finally, modeling examples are used as an instructional approach intended to support conceptual change processes by making effective SRL processes visible. These theoretical perspectives serve complementary roles within a unified conceptual framework.

The present study examines whether a video-based modeling intervention can promote facets of students’ SRL competence and their use of SRL strategies. Based on conceptual change theory, we assume that prior SRL competence influences both strategy use and responsiveness to instruction.

To address this overarching aim, this study proceeds in three analytical steps. First, we examine how different facets of SRL competence relate to students’ SRL strategy use and task performance. Second, we investigate whether a video-based modeling intervention improves these facets and students’ SRL strategy use. Third, we explore whether learners’ prior SRL competence moderates the effectiveness of the intervention.

By integrating SRL misconceptions with process-based measures, this study examines how learners’ beliefs, knowledge, and motivational orientations jointly relate to SRL behavior in complex tasks. To operationalize these aims, we formulate research questions corresponding to the three analytical steps outlined above.

Based on this framework, we address three sets of research questions aligned with the analytical steps outlined above. RQ3 represents the central research question of this study, whereas RQ1–RQ2 provide the theoretical basis for understanding SRL strategy use and performance. RQ4 explores whether learners’ prior competence influences the effectiveness of the intervention.

(1) Predictors of SRL behavior and performance:

RQ1. Which facets of SRL competence predict students’ reported SRL strategy deployment?

RQ2. Which facets of SRL competence predict students’ performance on a problem-solving task?

(2) Intervention effects:

RQ3. Does an intervention with video-based modeling examples lead to an improvement in facets of SRL competence and the use of reported SRL strategies?

(3) Moderation:

RQ4. Do prior facets of SRL competence moderate the learning of SRL?

RQ3 represents the central research question of this study, whereas RQ1 and RQ2 provide the theoretical basis for understanding how facets of SRL competence relate to strategy use and performance. RQ4 explores whether learners’ prior competence influences the effectiveness of the intervention.

## 2. Materials and Methods

### 2.1. Sample

#### 2.1.1. Pre- and Post-Intervention

The sample that participated in the laboratory part of this study (pre- and post-intervention) consisted of *N* = 157 students at a German university (*n* = 92 pre-service teachers, *n* = 19 psychology students, *n* = 14 educational sciences students, *n* = 26 sociology students, *n* = 6 not specified). One student had to be excluded from the sample due to too much missing data. Average participant age was *M* = 22.66 (*SD* = 3.71). A total of 81% were female. Their average semester of study at the time was *M* = 4.66 (*SD* = 3.07). See Table 1 for an overview of demographic variables per experimental condition.

#### 2.1.2. Follow-Up Assessment

Some participants were lost to follow-up four weeks after the initial laboratory study. *N* = 140 participants took part in all three measurement points (*n* = 82 pre-service teachers, *n* = 15 psychology students, *n* = 14 educational sciences students, *n* = 23 sociology students, *n* = 6 not specified). Average participant age was *M* = 22.54 (*SD* = 3.40). A total of 84% were female. Their average semester of study at the time was *M* = 4.57 (*SD* = 3.01). For a detailed breakdown of demographic variables by experimental condition during the follow-up assessment, please refer to Table S1 in Supplementary Materials S1, similar to the demographics presented for the full sample earlier.

### 2.2. Design

**Figure 1:**
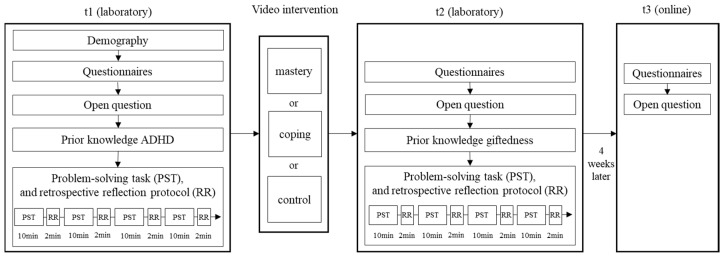
Study design. Note. t1–t3 = first, second, and third points of assessment, PST = problem-solving task, and RR = retrospective reflection.

Figure 1 illustrates the study design with three measurement points and three groups. Pre- and post-intervention data were assessed in the laboratory, with follow-up data gathered online four weeks later. Participants were recruited via student mailing lists, on-campus flyers, social media, and by advertisement in university seminars and lectures. Participants received compensation for their involvement. This study design received approval from the institute’s ethics commission, and participants provided informed consent prior to participation.

#### 2.2.1. Pre-Intervention

Before the intervention, participants answered questionnaires and an open question about facets of their SRL competence. Afterwards, they went to work on a complex problem-solving task on the topic of ADHD, with which we assessed their reported use of SRL strategies by means other than self-report questionnaire. All laboratory sessions were conducted individually. Participants completed this study in one-on-one sessions in a laboratory setting to ensure standardized testing conditions and to prevent interaction with other participants.

**Problem-Solving Task.** We first assessed participants’ prior knowledge of ADHD. Participants were then tasked with a 40-min problem-solving assignment where they had to prepare a parent–teacher talk about a student displaying symptoms of Attention Deficit Hyperactivity Disorder (ADHD). This involved informing parents about ADHD causes, symptoms, diagnosis, and implications, and relating this to the child’s behavior. To complete the task effectively, participants were given a significant amount of material with information about ADHD and observation of the child’s behavior that they could find on their desks in the laboratory. These materials consisted of the task description itself, books, papers, and excerpts from book chapters. Some of these materials contained information relevant to the task while others acted purely as distractors. These materials are detailed in Supplementary Materials S2. Given the task’s complexity and the volume of materials provided, participants were expected to employ SRL strategies in order to perform well on the task. For instance, they needed to first sift through and organize the materials to locate the task description hidden within them.

Participants were informed they could use all of the materials provided on their desk for the task and were required to document their notes on a computer in sufficient detail for others to use as a conversation guide. They were informed that they would automatically be forwarded to another page once the time was up. The remaining time was indicated by a countdown on the computer. Subsequently, participants’ notes on the task were coded to assess their task performance (see Supplementary Materials S3 for the coding scheme). Participants could reach up to 20 points in different categories, such as introduction to ADHD, the child’s diagnosis and consequences of ADHD. Prior to its use in this study, the task was piloted in a bigger sample of university students in one of our groups’ other projects.

Participants were periodically prompted by an audio signal every ten minutes to write down a retrospective reflection protocol; i.e., to write down their thoughts and actions over the previous ten minutes. They were instructed to write down everything that came to their mind related to the task, without any constraints on content, emphasizing that there were no right or wrong answers. We specifically did not instruct participants to write down SRL behaviors or strategies that they performed within the past ten minutes. This approach captured a comprehensive view of their thoughts and actions related to the task without filtering based on preconceived notions of what constitutes SRL. This task provided a meta-level snapshot of participants reported work behavior and were able to rate by ourselves whether the behavior and thoughts described contained SRL strategies or not.

Furthermore, we not only assessed this retrospective reflection protocol once per participant, but in total four times in the pre-assessment, hence receiving a processual measure, which is very close to participants’ actual behavior. Each reflection session was limited to two minutes. Hence, participants worked four times for 2 minutes on the reflection protocols and 40 min on the task itself, leading to 48 min in total. The protocols were then coded for participants’ use of SRL strategies (detailed coding scheme in Section 2). Before working on the problem-solving tasks, participants were first given an example of what such notes might look like (Supplementary Materials S4). The example depicted an unrelated scenario, cooking pasta with tomato sauce, in detail, ensuring participants were not influenced to replicate specific SRL strategies observed in the example during their own task reflections.

#### 2.2.2. Intervention

After completing the ADHD problem-solving task, participants were randomly assigned to either the mastery model intervention group or the control group. Once sufficient participants were recruited for comparisons between these groups, we recruited participants for the coping model intervention group, resulting in a quasi-experimental^1^ study design. We did so because we conducted this study in the laboratory in the months following the loosening of COVID-19 restrictions and students were still reluctant to take part in studies in the laboratory. They then watched a video of either (1) a mastery model or (2) a coping model being filmed while working on a university task, or they were assigned to (3) the control condition. The videos were all about ten minutes long and featured the same model in the same setting. The only difference between them was the model’s actions and her spoken comments about what she was doing. While the two intervention videos focused on instructing metacognitive learning strategies, the control group video did not refer to any SRL strategies at all. Video scripts can be found in Supplementary Materials S5, and the original videos are available upon request from the corresponding authors.

**Intervention Videos.** The two intervention videos taught identical metacognitive learning strategies, such as goal-setting, planning, and self-reflection. Additionally, both videos addressed common misconceptions about SRL and provided refutations of these misconceptions. Therefore, the instructional content of the intervention videos was identical. The primary distinction between them was the model used: In the mastery video, the model demonstrated proficient self-regulated behavior from the outset, whereas in the coping model video, the actress initially struggles with SRL and gradually adapts her approach. By comparing these two videos, we aimed to determine which modeling type could more effectively correct learners’ misconceptions of SRL.

In both experimental group videos, we employed signaling techniques (e.g., Tannert et al., 2023) by displaying the strategies being used or discussed, in order to direct learners’ attention. We informed the participants about the benefits of signaling, including enhanced strategy retention and potential application in their own learning practices, a method known as informed signaling. Tannert et al. (2023) found that informed signaling, when compared to uninformed signaling, led to significantly better conceptual knowledge about learning strategies and their promotion.

**Control Group Video.** The control group video features a student teacher discussing her experiences during her training at an elementary school. Every day after school, she records a brief video recounting her activities.

#### 2.2.3. Post-Intervention

After watching the video, participants were again asked to fill out questionnaires and answer the open question about facets of their SRL competence. Following this, they proceeded to engage in another problem-solving task focused on giftedness. As before the intervention, we assessed their prior knowledge of giftedness. The instructions for the task were similar to those for the ADHD assignment, but with a different case involving a potentially gifted boy. Participants were provided with a large amount of informative and some distracting material about giftedness (Supplementary Materials S2). Prior to its use in this study, we piloted this task with a sample of university students (*n* < 10).

#### 2.2.4. Follow-Up Assessment

Four weeks later, participants were asked to fill out an online survey, in which they completed the same questionnaires and open-ended questions about facets of their SRL competence as in the pre- and post-assessments.

### 2.3. Instruments

Data were assessed via questionnaire, a question with an open-ended answering format, two problem-solving tests focusing on educational scenarios related to ADHD and giftedness, and by a process-based retrospective reflection protocol.

#### 2.3.1. SRL Strategy Knowledge

SRL strategy knowledge was assessed by asking participants how they would describe SRL in their own words by means of an open question. Participants were prompted to take several minutes to reflect on their answer and to write down everything that came to their mind with regards to SRL. As synonyms for SRL, we also listed learning to learn and self-directed learning. Participants’ answers were then rated by two coders using a coding scheme developed by Dignath and Sprenger (2020). The coding scheme differentiates between the forethought, monitoring, and reflection phase and lists SRL behaviors that learners can perform during these stages. For example, goal-setting is listed as a strategy in the forethought phase, while comparison with objective is grounded in the reflection phase. More information on the coding scheme can be found in Dignath and Sprenger (2020). Before and during coding, the coders received 10 h of coder training. Interrater reliability was calculated beforehand based on 23% of the answers and proved to be satisfying (κ = 0.70, Landis & Koch, 1977). Therefore, the rest of the open answers were divided between the two coders and coded separately. Discrepancies were solved by discussion. A sum score was calculated for the nine SRL strategies the coding scheme contains. Therefore, scores on this measure could range between 0 and 9. Coders were blind to participants’ experimental conditions during the coding process to prevent potential bias in evaluating participants’ responses.

#### 2.3.2. Beliefs About SRL

Beliefs about SRL were assessed by a short version of the Beliefs about Teaching and Learning (BALT) questionnaire (Darmawan et al., 2020). The short version consists of 31 items with a six-point Likert scale ranging from *completely disagree* (1) to *completely agree* (6). The instrument simultaneously assesses beliefs that are consistent with SRL theory (14 items) and beliefs that are inconsistent with SRL theory (17 items). Both sub-scales showed satisfactory internal consistency across all measurement points (consistent beliefs: McDonald’s Ω_t1_ = 0.89, Ω_t2_ = 0.93, Ω_t3_ = 0.91; inconsistent beliefs_t1_: McDonald’s Ω_t1_ = 0.85, Ω_t2_ = 0.91, Ω_t3_ = 0.93). Beliefs inconsistent with SRL theory were treated as a separate construct representing misconceptions about SRL and were therefore not reverse-coded for the statistical analyses.

#### 2.3.3. Reported Strategy Use

Students’ reported strategy use was assessed by two means: the German Learning Strategies Inventory (LIST questionnaire, Schiefele & Wild, 1994), and by coding students’ written retrospective reflection protocols about their thoughts and actions during the problem-solving tasks with a coding scheme by J. H. Greene and Azevedo (2009). There is an ongoing discussion in the scientific SRL community as to whether SRL strategy use can be validly assessed with self-report questionnaires (Roth et al., 2016). Hence, we wanted to assess SRL strategy use not only by questionnaire, but also with another innovative approach that builds on thinking aloud protocols (J. A. Greene et al., 2011). Following the ongoing debate in the community, we believe that we assess different facets of learners’ SRL use with these two instruments. While a questionnaire provides us with information about students’ perception of themselves as self-regulated learners, which might not necessarily correspond to reality, the retrospective reflection protocol which we describe in more detail below is much closer to students’ SRL reality.

**German Learning Strategies Inventory.** We used the LIST questionnaire (German: Inventar zur Erfassung von Lernstrategien im Studium; Schiefele & Wild, 1994), a commonly used instrument in German-speaking countries (Spörer & Brunstein, 2006), which is based on the Motivated Strategies and Learning Questionnaire (MSLQ) by Pintrich et al. (1991). It claims to assess learning strategies students use for university studies. In the present study, we used 16 items from the scale *metacognitive strategies* and 4 items from the *time management* component of the subscale *resource-oriented strategies*. The LIST uses a 5-point Likert-scale ranging from *very rarely* (1) to *very often* (5). The reliability proved to be satisfactory: McDonald’s Ω_t1_ = 0.73, Ω_t2_ = 0.86, Ω_t3_ = 0.85. As described above, with this questionnaire measure, we aimed not to assess students’ actual SRL use, but rather their concept of themselves as self-regulated learners and their own perception of whether they would use a specific SRL strategy or not.

**Retrospective Reflection Protocol.** During their work on the problem-solving tasks, participants engaged in retrospective reflection protocols alongside note-taking for the parent–teacher talks. Every ten minutes, they were prompted to record their thoughts and actions within a two-minute time frame, generating a total of two-times-four reflection protocols per participant.

We used J. H. Greene and Azevedo’s (2009) coding scheme for micro- and macro-SRL processes, adapting it to our specific task (see Table 2 for a summary of codes and Supplementary Materials S6 for the full adapted coding scheme). Coders underwent 12 h of coder training before and during the coding process. Interrater reliability was initially assessed on 13% of the responses, found to be substantial (κ = 0.64 Landis & Koch, 1977), and was further improved during coding with an additional 13% sample (κ = 0.74). Thereafter, one coder analyzed the remaining protocols, resolving discrepancies through discussion.

We combined the macro-processes of planning and monitoring to indicate participant use of metacognitive SRL strategies, but only used micro-codes that reflected participants’ active use of SRL strategies, since we focused solely on active strategy use in the video intervention. Furthermore, we used the macro-process strategy to reflect participants’ use of cognitive strategies, but again only used sub-codes that indicated participants’ active use of strategies. By active use of strategies, we mean we only used sub-codes in the analysis that are indicative of participants’ strategic approaches to the learning task. For instance, codes such as *reading* and *re-reading* were not considered, as they do not inherently reflect strategic engagement when participants are unsure how to proceed.

This innovative and situational measure provides a much closer approximation of students’ actual SRL use compared to assessing SRL via questionnaire. By prompting students to report their thoughts and actions from recent minutes, we aimed to mitigate memory issues without disrupting their performance on the task by requiring immediate verbalization of their thoughts.

#### 2.3.4. Self-Efficacy Beliefs to Use SRL Strategies

Students’ self-efficacy beliefs to use SRL strategies were assessed by a modified version of the LIST (Schiefele & Wild, 1994), consisting of 20 items. We adapted the LIST items in a way that they do not assess strategy use, but self-efficacy beliefs in using strategies. For example, the original LIST item “I think about the order in which I work through the material beforehand” was adapted to “I am able to think about the order in which I work through the material beforehand.” We kept the original 5-point Likert scale, but used different scale labeling than the LIST, ranging from *cannot do at all* (1) to *highly certain can do* (5). The adapted instrument is displayed in Supplementary Materials S7. The reliability proved to be satisfactory: McDonald’s Ω_t1_ = 0.89, Ω_t2_ = 0.91, Ω_t3_ = 0.91.

#### 2.3.5. Utility of SRL Strategies

Students’ perceived utility of learning strategies was also assessed by a modified version of the LIST (Schiefele & Wild, 1994), also consisting of an adaptation of the 20 items. We adapted the LIST items so that they assess students’ beliefs about the utility of using SRL strategies. For example, the above-mentioned original LIST item was adapted to “I find it useful to think about the order in which I work through the material beforehand.” The scale labeling of the 5-point Likert scale was changed to *do not agree at all* (1) to *absolutely agree* (5). The adapted instrument is displayed in Supplementary Materials S7. The reliability proved to be satisfactory: McDonald’s Ω_t1_ = 0.81, Ω_t2_ = 0.89, Ω_t3_ = 0.87.

#### 2.3.6. Performance Measure

As described above, during the problem-solving tasks, participants were asked to take notes that were detailed enough to serve as a conversation guide for another person. For each task, these notes were scored by two coders using a coding scheme that we developed specifically for this study (see Supplementary Materials S3 for the coding schemes for both tasks). Participants could reach up to 20 points per task. Coders received detailed, 6 h-long coding training beforehand. The intraclass correlation coefficient was calculated based on 19% of the answers and proved to be good for the ADHD (κ = 0.82, Koo & Li, 2016) and excellent for the giftedness task (κ = 0.92). Therefore, the rest of the performance notes were divided between the coders and coded separately.

#### 2.3.7. ADHD and Giftedness Prior Knowledge

We also assessed participants’ prior knowledge about ADHD and giftedness. We used two questions with an open-ended answering format from the COACTIV-R study (Voss et al., 2011). Concerning ADHD, participants were asked to name the three core symptoms of ADHD, and concerning giftedness, participants were asked to list support measures for gifted children that they were aware of. Participants’ answers were then coded with the coding scheme from the COACTIV-R study. Participants could reach up to four points for both questions. Prior knowledge about ADHD and giftedness was assessed to control for participants’ domain-specific knowledge that might influence their performance on the problem-solving tasks. These variables were included as control variables in the regression analyses predicting task performance. Participants were not excluded based on prior knowledge levels because the tasks were designed to provide sufficient learning material for participants with varying levels of prior knowledge.

### 2.4. Analyses

We first conducted regression analyses in order to investigate whether facets of participants’ SRL competence predicted their reported use of SRL strategies (RQ1), and their performance on the problem-solving tasks (RQ2), including all predictors simultaneously. We then employed repeated measures ANOVAs to examine the impact of the intervention on participants’ SRL competence facets and reported SRL strategy use (RQ3). In cases where the normal distribution assumption was violated, we calculated pre–post-difference values of the individual variables and tested for significant differences between the groups using the Mann–Whitney *U*-test. We used G*Power (https://www.psychologie.hhu.de/arbeitsgruppen/allgemeine-psychologie-und-arbeitspsychologie/gpower, accessed on 17 March 2020) to conduct a priori sample size calculations based on Theobald’s (2021) work that found a mean effect size of 0.315 for interventions that focused on students’ metacognition to obtain 0.80 power at the standard 0.05 alpha error probability. The a priori sample size analysis detected a required sample size of 123 participants. As we found no significant differences between the mastery and the coping intervention groups using repeated measures ANOVA (for variables where the normality assumption was not violated) or Mann–Whitney U-test with the pre–post-difference scores (for variables where the normality assumption was violated, see Supplementary Materials S8), we combined them into one intervention group in all further analyses for greater power. Finally, we conducted exploratory analyses examining whether participants’ initial facets of SRL competence moderated the effectiveness of the video-based modeling examples using three-way interactions between the groups, the point of time, and specific facets of SRL competence at the pre-assessment (RQ4). We used FIML to deal with missing data (*n* = 2), which was missing completely at random.

### 2.5. Transparency and Openness

We report how we determined our sample size, all data exclusions, and all manipulations. All data, analysis code, and research materials are available upon request from the corresponding authors. Data were analyzed using R, version 4.3.1 (R Core Team, 2023). This study’s design and part of its hypotheses and analyses were pre-registered prospectively, before data were collected. More specifically, we pre-registered research questions. We did not pre-register research questions 1 and 2. However, since the data were available and the analyses could potentially lead to results interesting to the scientific community, we decided to further test research questions 1 and 2.

## 3. Results

SRL competence and students’ reported strategy use. Second, we analyze the effects of the intervention on SRL competence facets. Third, we explore whether prior SRL competence moderates these intervention effects.

### 3.1. Descriptive Statistics and Correlations

Table 3 shows the descriptives of all variables in the analyses, divided into the intervention and control group and the three measurement points. Participants in the pre-assessment in general had low SRL strategy knowledge (between 1.69 and 1.92 on a scale in the range of 0–9). They scored high on beliefs consistent with SRL theory, and low on beliefs inconsistent with SRL theory. Their self-efficacy beliefs were above scale mean, and their utility beliefs tended to be slightly higher. On average, they used metacognitive strategies in the range of 6.98–7.91 and cognitive strategies in the range of 7.92–8.33 in the ADHD problem-solving task.

Table 4 shows the scale intercorrelations (within the same point of time and between points of time). Overall, there was a trend towards positive significant correlations between all facets of SRL competence and reported SRL strategy use as assessed by the LIST and the performance on the problem-solving tasks, except for beliefs inconsistent with SRL theory, which were mainly correlated negatively with the other variables. Interestingly, participants’ knowledge of SRL did not correlate significantly with their self-efficacy beliefs, their utility beliefs or their reported SRL strategy use as assessed by the LIST. Furthermore, reported SRL strategy use as assessed by the LIST did not correlate significantly either with participants’ metacognitive and cognitive strategy use in the problem-solving task nor with their performance on this task. Due to high intercorrelations between reported metacognitive and cognitive strategy use in the problem-solving tasks across all time points and realizing these were not sufficiently independent, we created a composite SRL strategy use score by summing participants’ reported metacognitive and cognitive strategy use in the problem-solving tasks. Hence, this composite variable indicates how many SRL strategies students reported to use both before and after the intervention, which we utilized for all further analyses.

### 3.2. Structural Relations Between SRL Competence and Strategy Use

To provide a coherent analytical structure, the results are presented in three steps. First, we examine the structural relations between facets of SRL competence and students’ reported strategy use. Second, we analyze the effects of the intervention on SRL competence facets and strategy use. Third, we explore whether prior SRL competence moderates the effects of the intervention.

#### 3.2.1. Predicting Self-Reported SRL Strategy Use (LIST)

We used multiple regression to test which facets of SRL competence predict participants’ reported SRL strategy deployment. We conducted two separate analyses for the outcome variables reported SRL strategy use as assessed (a) by the LIST and (b) in the problem-solving task. We assume that the latter is closer to participants’ actual SRL behavior, while the former rather displays participants’ perception of their SRL behavior, which is not necessarily true to reality. As predictors, we included the variables (1a) learners’ beliefs consistent and (1b) inconsistent with SRL theory, (2a) their self-efficacy beliefs to use SRL, (2b) their utility beliefs about SRL strategy use, and (3) their knowledge of metacognitive learning strategies. We further modeled the interaction between self-efficacy and utility beliefs as suggested by the expectancy–value theory (Wigfield & Eccles, 2000).

Our results show that participants’ reported SRL strategy use as assessed by the LIST is predicted both by their self-efficacy beliefs, β = 0.19, *p* < 0.001, as well as by their utility beliefs, β = 0.18, *p* < 0.001 (see Table 5 for detailed results of all regression analyses). The Variance Inflation Index (VIF) indicated that there was no multicollinearity (Kennedy, 1992). The model explained 50% of the variance.

**Figure 2:**
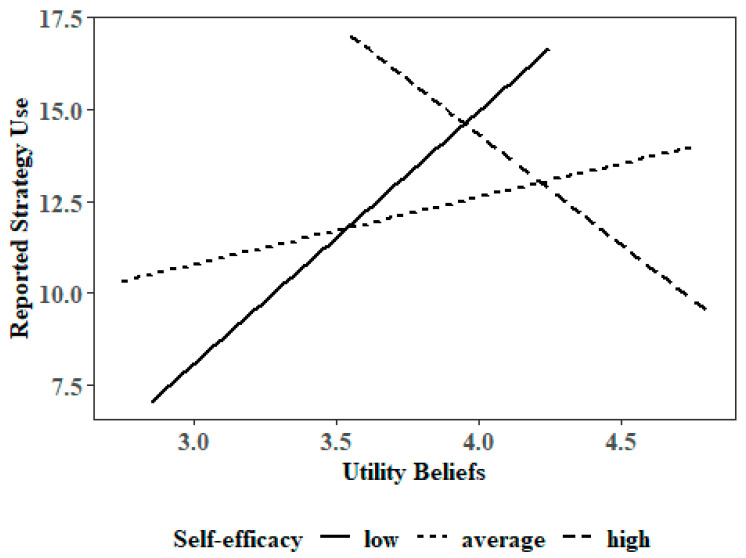
Interaction of learners’ self-efficacy and utility beliefs for their reported SRL strategy use.

Participants’ reported use of SRL strategy use in the problem-solving task was negatively predicted by their beliefs inconsistent with SRL theory, β = −0.25, *p* = 0.004. Furthermore, we found a significant interaction of self-efficacy and utility beliefs, β = −0.17, *p* = 0.04 (see Figure 2). While learners with low to average self-efficacy beliefs tend to report to use more SRL strategies in the problem-solving task when they have more utility beliefs, learners with a high self-efficacy tend to report less SRL strategies with increasing utility beliefs. There was no multicollinearity in the model and the variables resolved a moderate part of the variance (14%).

#### 3.2.2. Predicting SRL Strategy Use in the Problem-Solving Task

We again used multiple regression to test which facets of SRL competence predicted participant performance on the problem-solving tasks. We conducted separate analyses for the two problem-solving tasks: (a) ADHD and (b) giftedness. As predictors, we included the variables: (1a) learners’ beliefs consistent and (1b) inconsistent with SRL theory; (2a) their self-efficacy beliefs to use SRL; (2b) their utility beliefs about SRL strategy use; (3) their knowledge of metacognitive learning strategies, as well as (4a) their reported SRL strategy use as assessed by LIST; (4b) their reported SRL strategy use as coded from the retrospective reflection protocol; and either (5a) their prior ADHD or (5b) giftedness knowledge as control variables. Again, we also modeled the interaction between self-efficacy and utility beliefs. For the analysis of the ADHD task (which took place in the pre-assessment), we used the predictor variables assessed in the pre-assessment, while for the giftedness task that took place in the post-assessment, we used the predictor variables assessed in the post-assessment.

The results show that participants’ performance on the ADHD task was negatively predicted by their beliefs inconsistent with SRL theory, β = −0.22, *p* = 0.01, and positively predicted by their self-efficacy beliefs, β = 0.20, *p* = 0.04; their knowledge, β = 0.17, *p* = 0.032; and their reported use of SRL strategies in the problem-solving task, β = 0.27, *p* < 0.001. The VIF suggested that there was no multicollinearity in the model (Kennedy, 1992). The model explained 27% of the variance. Participants’ performance in the giftedness problem-solving task was predicted by their reported use of SRL strategies assessed in the problem-solving task, β = 0.32, *p* < 0.001, as well as their beliefs inconsistent with SRL theory, β = −0.19, *p* = 0.03. The models’ VIF again showed no multicollinearity (Kennedy, 1992). The variables resolved 27% of the variance, which is substantial (Cohen, 1988).

Beliefs inconsistent with SRL theory are particularly relevant for students’ actual strategic behavior in complex learning tasks.

### 3.3. Effects of the Video Intervention on SRL Competence

Next, we examined whether the modeling intervention was associated with changes in learners’ SRL competence facets and reported strategy use. These analyses compare changes between the intervention and control groups across the measurement points.

To examine whether participants in the intervention condition showed changes in facets of SRL competence as well as their use of SRL strategies, we conducted repeated measures ANOVAs or Mann–Whitney U-tests using differential scores from pre-to-post-assessment for variables where the normality assumption was violated, respectively. We conducted these analyses with the outcome variables (1a) learners’ beliefs consistent and (1b) inconsistent with SRL theory; (2a) self-efficacy beliefs to use SRL; (2b) utility beliefs about SRL strategy use; (3) knowledge of metacognitive learning strategies, as well as (4a) reported SRL strategy use as assessed by the LIST; (4b) reported SRL strategy use in the problem-solving task; and (5) performance in the problem-solving tasks. When the ANOVA revealed significant interaction effects, we also performed post hoc analyses (pairwise *t*-tests) with Bonferroni correction (see Supplementary Materials S8).

#### 3.3.1. Changes in SRL Beliefs

**Figure 3:**
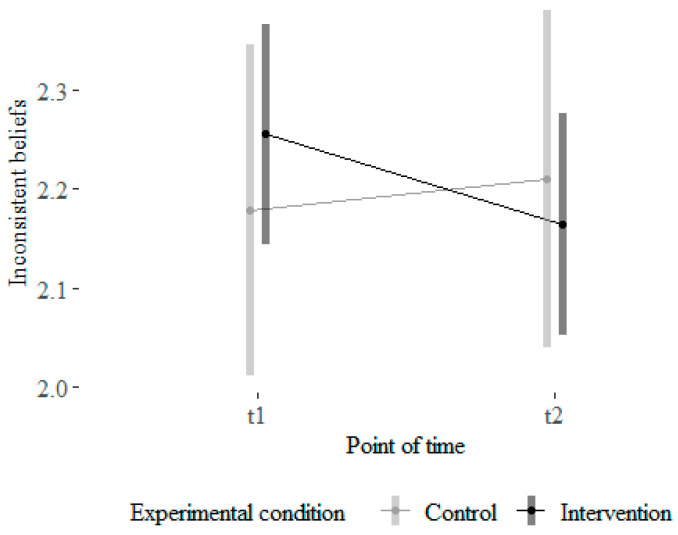
Participants’ beliefs inconsistent with SRL theory at the pre- and post-assessment. Note. t1 = pre-assessment; t2 = post-assessment.

For beliefs consistent with SRL theory as well as for beliefs inconsistent with SRL theory, one extreme outlier was identified and removed before performing the analyses. For both variables, the normality assumption was violated. Mann–Whitney U-tests with the pre–post-difference scores did not detect a significant difference in differential values for beliefs consistent with SRL theory. However, Mann–Whitney U-tests did detect a significant difference in differential values for beliefs inconsistent with SRL theory, W = 3084, *p* = 0.02). The beliefs inconsistent with SRL theory were reduced on a descriptive level in the intervention group, Mpre = 2.44, Mpost = 2.16, with a weak to medium effect size, r = 0.18 (Cohen, 1988), see Figure 3.

#### 3.3.2. Changes in Self-Efficacy and Utility Beliefs About SRL

For learners’ self-efficacy beliefs and their utility beliefs, the normality assumption held up. Repeated measures ANOVAs did not detect any significant main effect of group, point of time, or the interaction of the two for either variable.

#### 3.3.3. Changes in Strategy Knowledge

**Figure 4:**
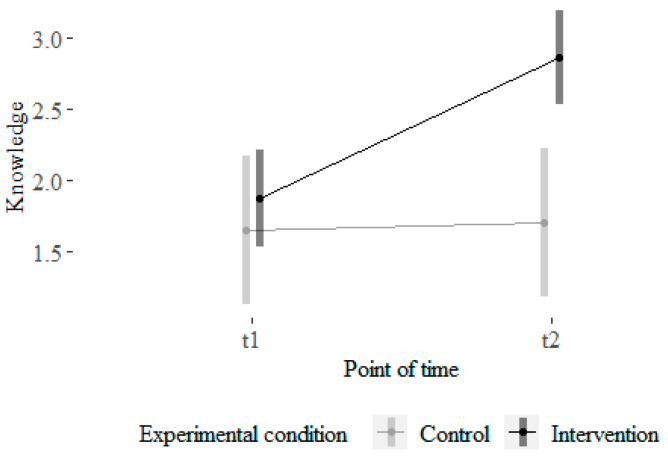
Participants’ SRL strategy knowledge at the pre- and post-assessment. Note. t1 = pre-assessment; t2 = post-assessment.

For learners’ SRL strategy knowledge, the normality assumption was violated. Hence, we conducted the Mann–Whitney U-test for differential scores, which detected a significant difference in differential values for participants’ knowledge between the intervention and the control group (W = 1750, *p* = 0.002). Participants in the intervention group showed higher post-intervention knowledge scores compared to the control group, Mpre = 1.93, Mpost = 2.85, with a medium effect size, r = 0.25 (Cohen, 1988) (see Figure 4).

#### 3.3.4. Learners’ Reported Use of SRL Strategies

For learners’ reported use of SRL strategies as assessed by the LIST, which was not normally distributed, and for learners’ reported SRL strategy use in the problem-solving task, which was normally distributed, repeated measures ANOVAs or Mann–Whitney U-test did not detect a significant main effect of group, point of time, or the interaction of the two.

#### 3.3.5. Learners’ Performance on the Problem-Solving Tasks

Finally, for learners’ performance on the problem-solving tasks, which was not normally distributed, we again conducted Mann–Whitney U-test, which did not find a significant difference between the groups’ differential values.

#### 3.3.6. Changes in Facets of Learners’ SRL Competence and Reported SRL Strategy Use Towards the Follow-Up Assessment

For all variables, we additionally checked whether there were significant changes from post- to follow-up assessment. However, we did not find such significant changes (see Supplementary Materials S8, Table S3 for detailed results).

### 3.4. Moderation of Intervention Effects by Prior SRL Competence

Finally, we examined whether the effectiveness of the intervention depended on learners’ initial SRL competence. Specifically, we tested whether prior SRL strategy use moderated the effects of the intervention on subsequent strategy use.

In order to check whether learners’ prior facets of SRL competence moderate the learning of facets of SRL competence and reported strategy use, we extended the repeated measures ANOVA models from RQ3 and added the facets of SRL competence as well as participants’ reported SRL strategy use at the pre-test as moderators in these models. We found a significant three-way interaction for the self-reported use of SRL strategies as assessed by the LIST, F(1, 306) = 4.83, *p* = 0.03, η 2G = 0.02. Post hoc analysis revealed a strong significant difference in the increase in use of SRL strategies from pre- to post-assessment between the two groups, t = 2.44, df = 306, *p* = 0.02 (Cohen’s d = 0.83; Cohen, 1988) for participants with low prior SRL use.

**Figure 5:**
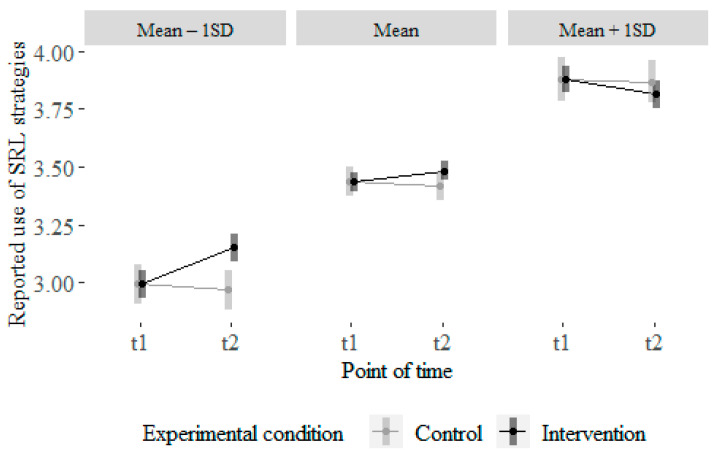
Significant three-way interaction between group, point of time, and use of SRL strategies. Note. t1 = pre-assessment, t2 = post-assessment, and SD = standard deviation.

Figure 5 shows how students benefited from the videos according to their reported prior use of SRL strategies: students who reported low to average prior use of SRL strategies (left column and middle column) benefited from the intervention, whereas students who reported using a lot of SRL strategies at the pre-assessment did not report to benefit from the intervention.

Taken together, the results suggest that learners’ beliefs about SRL play a central role in shaping their strategy use, and that the intervention particularly benefited learners with initially lower levels of SRL strategy use.

## 4. Discussion

SRL is a core competence that plays an important role in academic studies. It is therefore crucial to know which SRL skills predict students’ SRL and how these can be promoted. Based on Winne and Hadwin’s COPES model (1998), we looked into which facets of SRL competence, including (1a) learners’ beliefs consistent and (1b) inconsistent with SRL theory, (2a) their self-efficacy beliefs to use SRL as well as (2b) their utility beliefs about SRL strategy use, and (3) their knowledge of metacognitive learning strategies, predict their reported use of SRL strategies as well as their achievement. Different from many other authors in this field, we looked at multiple facets of SRL competence simultaneously. This procedure has several advantages: (1) Given the interrelated nature of SRL competence facets, their mutual influence warrants a simultaneous analysis to discern which facets remain significant when all are considered together. This approach enables a comprehensive understanding of their predictive power. (2) By incorporating all facets into a single model and standardizing the data, our regression analyses identify the most robust predictor of reported SRL strategy use and performance. This highlights the facet warranting focused attention. (3) Recognizing the interplay among SRL facets underscores the importance of profiling learners’ SRL competence to comprehend their strengths and weaknesses. Our data enable the identification of specific areas where individuals and groups may require targeted support. While these analyses extend beyond the current article’s scope, we plan to address them in a forthcoming publication.

We assessed participants’ reported use of SRL strategies in a multi-method way by means of a self-report questionnaire, which depicts their own perception of their SRL strategy use, and by means of thinking-aloud data resulting from a retrospective reflection protocol, which participants created during working on two complex problem-solving tasks and which is closer to participants’ actual SRL behavior. Furthermore, we investigated the predictors of participants’ performance on the two problem-solving tasks we used in the pre- and post-assessments. Finally, we used video-based modeling examples to improve facets of participants’ SRL competence and their SRL strategy use and tested whether learners’ initial facets of SRL competence influenced how much they learned from the intervention. Our analyses revealed five cornerstone findings:

**1. Adopting a Facet-Based, Competency-Driven Approach Proves Valuable in Predicting SRL Strategy Use.** We found that participants’ reported use of SRL strategies as assessed by the LIST questionnaire was positively predicted by their self-efficacy to use SRL strategies, which is in line with findings by Cerezo et al. (2019), as well as by students’ utility beliefs about SRL strategies. This is contrary to Cerezo et al.’s (2019) finding that utility beliefs did not predict students’ use of SRL strategies. Furthermore, participants’ beliefs, which were inconsistent with SRL theory, negatively predicted their use of SRL strategies in the problem-solving task, which is consistent with Vosniadou et al.’s (2021) findings. Different from what we expected, participants’ beliefs, which are consistent with SRL theory, did not significantly predict their use of SRL strategies. Participants’ use of SRL strategies in this task was further predicted by the interaction of self-efficacy and utility beliefs, which on the first look is according to expectancy–value theory (Wigfield & Eccles, 2000).

However, upon closer inspection, we find that participants who have high self-efficacy and high utility beliefs tend to report less SRL strategy use, while participants with high self-efficacy and low utility beliefs tend to report more SRL strategy use. This is actually contrary to expectancy–value theory (Wigfield & Eccles, 2000). This finding might be explained due to a ceiling effect of the moderator variable self-efficacy beliefs: Overall, participants scored very high (M = 3.98 on a scale from 1–5) on this measure. When conducting a simple slopes analysis, participants are being split up into three different groups depending on their score on this moderator variable. However, with this ceiling effect, even participants in the low self-efficacy group actually held average self-efficacy beliefs (M = 2.87), while participants in the average self-efficacy group were actually already above scale mean (M = 3.56). For these two groups, the utility value hypothesis held up: with increasing utility beliefs, they also reported more SRL strategy use. However, the last group of high self-efficacy beliefs was actually almost overconfident in their ability to self-regulate (M = 4.31).

One possible interpretation is that for learners with very high self-efficacy beliefs, additional increases in perceived utility may not translate into increased strategy use, suggesting a potential saturation effect. In other words, once learners already feel highly capable of using SRL strategies, the perceived usefulness of these strategies may add little additional motivational impetus for their implementation. However, this interpretation should be considered cautiously, as the present study did not assess calibration between perceived competence and actual performance. Future research could examine whether discrepancies between perceived and actual competence contribute to such interaction effects.

Based on our findings, students’ self-efficacy beliefs in using SRL strategies, their beliefs about the utility of SRL strategies, and their beliefs inconsistent with SRL theory seem to play a role for their reported use of SRL strategies. Keeping in mind that most studies that looked into the relationship between facets of SRL competence and SRL strategy use used self-report questionnaires, the results based on the retrospective reflection protocol data are even more meaningful. It is therefore crucial to address diverse facets of SRL competence to enhance students’ strategic learning behaviors. Furthermore, this study is the first to address all facets of SRL competence simultaneously and hence highlighting the individual relative importance of each facet.

We did find different SRL competence facets to predict students’ SRL strategy use, depending on whether we assessed it with questionnaire or with retrospective reflection protocol. As we described earlier, we believe that we assess different things with the two measures: the self-report questionnaire provides us with an idea which perceptions learners have of themselves as self-regulated learners, while the retrospective reflection protocol is situational and delivers data that are closer to participants’ actual behavior. Hence, the finding that it is different facets that predict both comes natural to us.

**2. Facets of Learners’ SRL Competence and SRL Strategy Use Also Predict Their Performance.** This study is also the first to address all facets of SRL competence simultaneously with regard to students’ performance and hence highlighting the individual relative importance of each facet. In both the pre- and post-assessment, participants’ beliefs that were inconsistent with the SRL theory negatively predicted learners’ performance. These results were as expected and in line with Vosniadou et al.’s (2021) findings. It is also consistent with other findings (e.g., Broadbent & Poon, 2015; Dent & Koenka, 2015) that the (reported) use of SRL strategies positively predicted performance at both assessment points. The more SRL strategies participants reported in the retrospective reflection protocols, such as planning, organizing, setting goals, reflecting, highlighting, or summarization, the better their performance was on both tasks. Since we created a composite variable that contains both participants’ cognitive and metacognitive strategy use, no conclusions can be drawn as to which kind of strategy use better predicts students’ achievement.

However, in terms of student education, our results show that it is desirable to foster students’ general SRL strategy use and hence indirectly improve their performance. Like other papers (e.g., Artelt & Schneider, 2015; Chytrý et al., 2020; Soodla et al., 2017; Swanson, 1990; Tian et al., 2018), we further found a significant prediction of participants’ SRL strategy knowledge on their performance. The prediction of self-efficacy beliefs about SRL strategies for performance was also as expected (Schmidt et al., 2012; Zimmerman, 2013). Taken together, our results indicate that students’ beliefs that were inconsistent with SRL theory and their strategy use predicted their performance in both tasks. This finding brings us to our next cornerstone finding:

**3. SRL-Inconsistent Beliefs are Detrimental for SRL Strategy Use and Student Performance.** In regression analyses with both the outcomes SRL strategy use and performance, we found students’ beliefs inconsistent with the SRL theory to be an important negative predictor. However, contrary to our expectations and Vosniadou et al.’s (2021) findings, beliefs consistent with the SRL theory did not significantly predict learners’ achievement. We can therefore derive that rather than focusing on strengthening learners’ beliefs consistent with the SRL theory, one should pay close attention to assess their beliefs inconsistent with the SRL theory and then refute these misconceptions. The approach of distinguishing between consistent and inconsistent beliefs pays off and should be further pursued.

**4. A Knowing–Doing Gap Between Self-Efficacy and Actual SRL Strategy Use.** Another noteworthy finding concerns the discrepancy between predictors of self-reported SRL strategy use and predictors of strategy use observed in the problem-solving task. While self-efficacy beliefs significantly predicted students’ self-reported strategy use in the LIST questionnaire, they did not predict students’ reported strategy use during the task itself.

This pattern may reflect a “knowing–doing gap,” in which learners’ beliefs about their capabilities influence their self-perception as strategic learners but do not necessarily translate into actual strategy implementation in complex learning situations.

This relationship can also be explained by the nature of the problem-solving tasks. The performance tasks required participants to organize information, integrate evidence, and formulate explanations. These demands align particularly closely with SRL processes such as planning, monitoring, and strategic information search. Students who reported more frequent use of these strategies during the task were therefore better able to structure the available materials and produce more comprehensive solutions.

This finding further highlights the importance of situational measures of SRL processes, as self-report questionnaires may capture learners’ general beliefs about their learning behavior rather than their actual strategic behavior during specific tasks.

An important implication of our findings is that the different measures of SRL competence appear to capture distinct components of a broader SRL architecture rather than interchangeable indicators of the same construct. Strategy knowledge, beliefs about SRL, motivational beliefs, and observed strategy use were only weakly correlated, suggesting that each instrument captures different epistemic aspects of self-regulation. This pattern suggests that questionnaire-based measures may primarily capture learners’ beliefs about their typical study behavior rather than their actual strategic activity in complex learning situations. From an epistemic perspective, this finding highlights the importance of complementing questionnaire-based SRL assessments with process-based measures that capture learning behavior within specific contexts.

**5. Short and Economic Video Interventions Using Modeling Examples Can Elicit Conceptual Change in SRL.** Our results show that our video intervention significantly improved students’ knowledge of SRL and was further able to reduce participants’ beliefs inconsistent with SRL theory. This finding is especially promising since we also found that it is, amongst other facets of SRL competence, learners’ knowledge on SRL as well as their beliefs inconsistent with SRL theory that predict whether they (report to) use SRL strategies and how well they perform. Even more promising is the fact that the videos used in this study were rather short and economical, and could therefore be administered to a bigger audience with ease. This study was one of the first to look into how conceptual change could be attained in the field of SRL (Vosniadou et al., 2020).

**6. Students’ May Profit Differently From Interventions Depending on Their Prerequisites.** Lastly, only students who reported low to medium prior use of SRL strategies in the pre-assessment benefited from the intervention and showed an improvement in their self-reported SRL strategy use in the post-assessment, whereas students who reported high prior use of SRL strategies did not benefit from the intervention. Since this was assessed using a self-report, we do not think that learners’ SRL use has actually changed since before the pre-test. It is not plausible that learners’ use of SRL had already changed at this point in time, since they answered the questionnaire directly after a break after watching the videos. Therefore, they did not have the time to actually employ said strategies. Rather, we believe that with this measure we assessed learners’ concepts of themselves as self-regulated learners and assessed whether they think they would use SRL strategies in a learning situation. Therefore, this result likely does not depict learners’ actual behavior. However, it is still a promising result, since learners’ perception of their own SRL strategy use is likely closely related to their actual strategy use.

**7. Greater Gains for Low-SRL Learners Reflect the Expertise Reversal Effect.** Interestingly, our findings revealed that students with initially low to average SRL strategy use benefited most from the intervention. At first glance, this result appears to contradict our assumption derived from conceptual change theory that learners with more advanced prior conceptions may be better able to integrate new information. However, this pattern can also be interpreted in light of the expertise reversal effect (Kalyuga et al., 2003). According to this perspective, instructional supports that are beneficial for novices may become redundant for more knowledgeable learners. In the present study, the modeling videos may have provided particularly useful scaffolding for learners with limited prior SRL strategy use, whereas students who already reported frequent strategy use may have experienced fewer additional benefits or encountered a ceiling effect. From a conceptual change perspective, this finding may also suggest that learners with weaker initial SRL competence had more misconceptions or gaps in their understanding that could be addressed by the intervention. Consequently, these learners may have experienced stronger conceptual restructuring than learners whose conceptions were already relatively aligned with the SRL theory.

**8. Refutation Rather Than Modeling Type May Explain the Effects.** Contrary to our expectations, we did not find differential effects between mastery and coping modeling examples. One possible explanation is that both videos contained identical instructional content regarding SRL strategies and explicitly addressed misconceptions through refutation. Hence, the conceptual change processes may have been primarily driven by the refutation of misconceptions rather than by the type of model. Another explanation may relate to participants’ prior SRL competence. University students may already possess moderate familiarity with SRL concepts, which could reduce sensitivity to differences between coping and mastery models (Van Gog & Rummel, 2010). Additionally, the relatively short duration of the intervention video may have limited the opportunity for learners to deeply process differences between modeling types. Future research could investigate whether longer modeling sequences, stronger contrasts between model behaviors, or repeated exposure lead to stronger differentiation between mastery and coping modeling approaches.

**9. From Belief Change to Behavioral Change: A Gradual Process.** Our findings also provide partial support for the conceptual change perspective that guided this study. The intervention successfully reduced beliefs inconsistent with SRL theory and increased SRL strategy knowledge, suggesting that conceptual change regarding SRL is possible even through relatively brief interventions. However, changes in knowledge and beliefs did not translate into immediate changes in reported strategy use or performance. The present study primarily captures changes in learners’ SRL knowledge and beliefs rather than deeper conceptual restructuring processes. Consequently, the findings should be interpreted as evidence of shifts in SRL-related conceptions rather than definitive proof of conceptual change in the strict sense.

This finding aligns with conceptual change research suggesting that modifying prior beliefs and integrating new conceptions into behavior can be a gradual process (Vosniadou et al., 2008). Learners may acquire new conceptual understanding about SRL without immediately applying it in practice. Future research should therefore investigate longer-term interventions or repeated opportunities for practice that may facilitate the transfer of conceptual change into actual strategy use.

**10. Misconceptions About SRL Can Hinder Strategy Use and Performance.** The finding that beliefs inconsistent with SRL theory negatively predicted students’ strategy use and performance can also be interpreted from the perspective of conceptual change theory. Conceptual change research suggests that learners’ prior conceptions may act as filters through which new information is interpreted (Vosniadou et al., 2008). If learners hold misconceptions about SRL—such as the belief that strategies are only necessary in difficult situations—these beliefs may prevent them from applying strategies even when they possess relevant strategy knowledge. In this sense, misconceptions about SRL can function as cognitive barriers that hinder the translation of knowledge into action. Our findings are consistent with the assumption that promoting SRL may require not only teaching strategies but also explicitly addressing and revising learners’ prior beliefs about learning.

This interpretation is also consistent with the multidimensional conceptualization of SRL competence adopted in this study, according to which learners’ knowledge, beliefs, and motivational orientations jointly shape the deployment of SRL strategies.

### 4.1. Limitations and Recommendations for Future Research

Our research comprises some limitations. First, we are aware that self-reports can bias the assessment of SRL use (Winne & Perry, 2000) and therefore assessed reported SRL strategy use by multiple means. We believe that with the self-report questionnaire, we received a glimpse into participants’ perception of their own SRL practice, while with the retrospective reflection protocol, we received a measure that is closer to participants’ actual behavior. Therefore, we are more likely to trust the analyses with the retrospective reflection data. However, in the follow-up assessment, a self-report questionnaire was our only measure of SRL strategy use.

Secondly, the retrospective reflection protocol measure, in which students report on what they have been doing in the past ten minutes and what their thoughts have been during this period, is a snapshot of students’ use of SRL strategies. As we further aggregated students’ responses across all four measurement points in the pre- and the post-assessment, we only have a general assessment of students’ reported use of SRL strategies. Nevertheless, this measure includes four very specific learning situations that were recorded over a longer period of time during the process. In subsequent studies, we will look at the process of reported SRL strategy use with the data that we analyzed, for example to check for differences in students’ reported SRL strategy use at the beginning and end of the problem-solving tasks, with the help of process analyses. Furthermore, as the name itself suggests, a retrospective reflection protocol may trigger SRL processes by the mere use of this assessment method and act as an intervention itself, even though this was not the desired outcome from using this assessment method, and might impede students’ task performance (J. A. Greene et al., 2011).

Furthermore, the quasi-experimental nature of this study might reduce its internal validity. For example, selection bias might have led to an unequal distribution of participants to experimental groups. However, we did not find any significant differences between the mastery and control group with regards to their learning from the intervention videos. In addition, given the quasi-experimental design, causal conclusions about the intervention should be drawn with caution. The findings therefore indicate intervention-related changes rather than definitive causal effects.

Moreover, we found self-efficacy and utility beliefs to predict students’ reported use of SRL strategies as assessed by the LIST. This prediction might also be due to the fact that we assessed the two predictor variables with items that were derived from the LIST. However, multicollinearity in the models was satisfactory. Future research should further validate the adaptation of the LIST items and assess its convergent and discriminant validity in relation to other variables.

In addition to measurement-related considerations, some limitations of this study design should be acknowledged. The ADHD problem-solving task was always administered before the intervention and the giftedness task afterwards. This fixed order was necessary because the ADHD task served as the baseline measure of SRL strategy use prior to the intervention, whereas the giftedness task assessed SRL behavior after the intervention. Counterbalancing the order would have confounded the interpretation of intervention effects. However, we acknowledge that task order effects cannot be entirely ruled out and future studies could include additional parallel tasks to further control for potential sequence effects.

Moreover, in order to reduce multicollinearity between metacognitive and cognitive strategy use, we created a composite SRL strategy score combining both strategy types. While this approach allowed us to capture overall strategic engagement during the problem-solving tasks, it also limits the interpretation of our results. Specifically, it prevents conclusions about whether metacognitive strategies (e.g., planning or monitoring) or cognitive strategies (e.g., summarizing or elaborating) were more strongly related to performance. Although both types of strategies were observed in participants’ reflection protocols, future research could examine these strategy categories separately in order to better understand their differential contributions to learning outcomes. Such analyses may provide deeper insights into which specific SRL processes are most critical for successful problem-solving.

Beyond these design considerations, the scope of the present study should also be taken into account. The present study focused on SRL processes rather than domain-specific knowledge acquisition. While participants worked extensively with content related to ADHD and giftedness, we did not assess participants’ learning gains regarding these topics. Future research could examine whether improvements in SRL competence also translate into greater domain-specific learning outcomes.

Furthermore, our sample consisted of students of social sciences (student teachers, psychology, sociology, and educational sciences students). The results can therefore only be generalized to other target groups to a limited extent. Further studies could identify individual differences between different target groups and test differential effects for SRL interventions.

Also, the study that we conducted was very thorough and therefore took over three hours to complete in the laboratory (pre- and post-assessments combined). Although we reduced student fatigue by letting them take a break after the pre-assessment before watching the video, we are aware that their answers, especially after the intervention, might be influenced by this cognitively challenging study.

Finally, the quasi-experimental nature of this study should be acknowledged as a limitation. Due to the circumstances surrounding the COVID-19 pandemic, participants for the different experimental conditions were recruited during different phases of data collection. Although the study procedures were kept identical across conditions, external factors related to the broader educational context during this time may have influenced students’ motivation or learning behavior. Future studies using fully randomized designs conducted within a narrower time frame could help further rule out potential history effects.

### 4.2. Implications for Future Research and Practice

Taken together, the central contribution of this study lies in integrating research on SRL misconceptions with process-based measurement of strategy use. By examining how learners’ beliefs, knowledge, and motivational orientations jointly relate to observed SRL behavior during complex tasks, this study provides new insights into how misconceptions about SRL may hinder the translation of strategy knowledge into actual learning behavior.

We identified facets of SRL competence, namely students’ beliefs inconsistent with SRL theory, their knowledge of SRL, as well as their self-efficacy and utility beliefs, which predict learners’ reported SRL strategy use. Furthermore, we found that students’ beliefs inconsistent with the SRL theory, as well as their reported use of SRL strategies, their knowledge, and their self-efficacy beliefs, predicted their problem-solving performance. We thus propose a competency-based approach to SRL research, in which not only SRL strategies are the target of investigations and interventions, but also facets of SRL competence that are causal to the use of SRL strategies. Future research should therefore take into account the different SRL competence facets and not just focus on improving overall SRL as a whole, for example by focusing on students’ mastery and vicarious experiences or on social persuasion related to SRL, to foster their SRL self-efficacy (Usher & Pajares, 2006). Furthermore, it would be desirable that future research does not only look into whether single competence facets predict SRL strategy use, but also to look into even more possible interactions and relationships between these variables (other than the interaction between expectancy × value in this study). Due to the sample size, in this study it was not possible to model relationships between the facets with structural equation modeling or path analysis. However, this would be a worthwhile venture for future studies in the field.

Moreover, fostering aspects of students’ SRL competence with modeling videos seems to be a valuable pursuit to support conceptual change towards SRL. It is especially promising that these videos seem to be specifically good for students’ knowledge of SRL, as we found it predicted students’ performance, and students’ beliefs inconsistent with SRL theory, since we found it predicted students’ reported use of SRL strategies in the problem-solving task, as well as their performance in both the pre- and the post-assessment. As a cornerstone of future research agendas, it would be desirable to place even more emphasis on learners’ beliefs that are inconsistent with SRL theory, as our and other researchers’ findings (Vosniadou et al., 2021) show that these consistently negatively predict learners’ reported use of SRL strategies, and student performance.

Going one step further than to try to improve facets of students’ SRL competence and their reported SRL strategy use with modeling examples, future research could also focus in more detail on improving in-service teachers’ knowledge and reducing their beliefs that are inconsistent with SRL theory. This would be helpful, as teachers are in a good position to promote their students’ SRL and it is facets of teacher competences, such as their knowledge and beliefs that predict their SRL promotion. A better SRL promotion by teachers would in turn improve students’ SRL (e.g., Authors, under review; Fischer & Dignath, 2023; Paris & Winograd, 2003). Therefore, the promotion of facets of teacher SRL competence should indirectly improve students’ achievement via teachers’ better promotion of SRL.

Finally, particular attention should be paid to learners with low and medium reported prior SRL use, as they report to benefit more from such interventions. When designing interventions, future research could therefore take into account learners’ prerequisites in more detail, for example by creating slightly different versions of an intervention for students with different (self-reported) levels of prior use of SRL strategies. As an implication for practice, it could be assumed that students profit differently from their teachers’ SRL promotion depending on their prior familiarity with and use of SRL. It is therefore essential that teachers learn how to accurately diagnose their students’ SRL (Karlen et al., 2023), in order for them to be able to provide individualized promotion of SRL to every student.

## Figures and Tables

**Table 1:** Demographics of the three experimental conditions—pre- and post-intervention.

Variable	Mastery	Coping	Control	Total
*n*	56	53	48	157
Age	*M* = 23.00 *SD* = 3.63	*M* = 22.40 *SD* = 2.94	*M* = 22.60 *SD* = 4.52	*M* = 22.66 *SD* = 3.71
Semester	*M* = 4.89 *SD* = 3.44	*M* = 4.62 *SD* = 2.98	*M* = 4.44 *SD* = 2.73	*M* = 4.66 *SD* = 3.07
% Female	79	81	83	81
% Pre-service teachers	41	53	65	59
% Psychology	13	17	6	12
% Educational sciences	14	4	8	9
% Sociology	32	6	10	17

Notes. Percentages of subjects do not round up to 100% due to NAs.

**Table 2:** Overview of the codes from the retrospective reflection protocol.

Planning	Monitoring	Strategy Use	Task Difficulty and Demands	Interest
Orientation Planning Goals Prior knowledge activation Recycle goal in working memory	Judgment of learning Judgment of knowing Metacognitive awareness Self-questioning Content evaluation Evaluate content as answer to goal Monitor progress towards goals Monitor use of strategies	Selecting a new informational source Coordinating informational sources Review notes Memorization Free search Goal-directed search Summarization Taking notes Highlighting text passages Draw Inferences Hypothesizing Knowledge elaboration Mnemonic Find location in environment Skip	Time and effort planning Help seeking behavior Task difficulty Control of context Sorting, organizing, grouping Cleaning space	Interest statement (positive or negative)

**Table 3:** Descriptives of all variables.

		Intervention Group					Control Group				
Variable	Range	*M*	*SD*	Min	Max	*n*	*M*	*SD*	Min	Max	*n*
	Pre-assessment (*N* = 157)										
Knowledge	0–9	1.92	1.62	0	7	107	1.69	1.67	0	6	48
Consistent beliefs	1–6	4.97	0.59	3.21	6	109	4.94	0.42	3.93	5.79	48
Inconsistent beliefs	1–6	2.26	0.60	1.29	4.59	109	2.19	0.45	1.29	3.29	48
Self-efficacy beliefs	1–5	3.64	0.53	2.35	2.85	109	3.57	0.57	2.25	4.9	48
Utility beliefs	1–5	4.00	0.42	2.90	4.80	109	3.92	0.45	2.75	4.7	48
Reported SRL use (LIST)	1–5	3.45	0.44	2.20	4.60	109	3.41	0.45	2.25	4.5	48
Task performance ADHD	0–20	7.93	4.41	0	18	109	8.48	4.28	0	19	48
Reported metacognitive strategy use	0–∞	7.91	4.37	0	20	109	6.98	3.54	1	14	48
Reported cognitive strategy use	0–∞	7.92	4.59	0	17	109	8.33	4.64	1	18	48
	Post-assessment (*N* = 157)										
Knowledge	0–9	2.86	2.14	0	9	109	1.67	1.28	0	5	48
Consistent beliefs	1–6	4.96	0.67	2.71	6	109	4.93	0.50	4.00	6.00	46
Inconsistent beliefs	1–6	2.16	0.71	1.06	5.59	109	2.22	0.45	1.41	3.35	46
Self-efficacy beliefs	1–5	3.65	0.56	1.85	4.95	109	3.62	0.57	2.5	4.75	48
Utility beliefs	1–5	4.08	0.49	2.85	5.00	109	3.94	0.49	2.9	4.95	48
Reported SRL use (LIST)	1–5	3.49	0.47	2	4.75	109	3.39	0.51	2.45	4.55	48
Task performance giftedness	0–20	8.66	4.97	0	18	107	8.49	4.45	0	18	47
Reported metacognitive strategy use	0–∞	7.71	4.40	0	19	109	6.71	3.99	0	15	48
Reported cognitive strategy use	0–∞	6.65	4.43	0	18	109	7.21	4.40	1	18	48
	Follow-up assessment (*N* = 140)										
Knowledge	0–9	2.05	1.49	0	9	97	1.44	1.33	0	4	43
Consistent beliefs	1–6	5.04	0.58	3.14	6	96	4.85	0.52	3.50	6.00	43
Inconsistent beliefs	1–6	2.40	0.83	1.12	5.24	96	2.47	0.75	1.35	5.00	43
Self-efficacy beliefs	1–5	3.71	0.52	2.30	5.00	96	3.55	0.50	2.35	4.55	43
Utility beliefs	1–5	4.10	0.43	2.70	4.85	96	3.90	0.45	2.75	4.80	43
Reported SRL use (LIST)	1–5	3.59	0.44	2.60	4.55	96	3.43	0.49	2.30	4.35	43

Notes. SRL = self-regulation of learning.

**Table 4:** Intercorrelations of variables within and between time points.

	Scale		Zero-Order Correlations								
			1	2	3	4	5	6	7	8	9
1	Knowledge	(t1/t1)	-								
		(t2/t2)	-								
		(t3/t3)	-								
		(t1/t2)	0.51 ***								
		(t1/t3)	0.52 ***								
		(t2/t3)	0.59 ***								
2	Consistent beliefs	(t1/t1)	0.12	-							
		(t2/t2)	0.20 *	-							
		(t3/t3)	0.27 **	-							
		(t1/t2)	0.09	0.74 ***							
		(t1/t3)	0.19 *	0.59 ***							
		(t2/t1)	0.22 **	-							
		(t2/t3)	0.24 **	0.62 ***							
		(t3/t1)	0.16	-							
		(t3/t2)	0.18 *	-							
3	Inconsistent beliefs	(t1/t1)	−0.25 **	−0.40 ***	-						
		(t2/t2)	−0.29 ***	−0.37 ***	-						
		(t3/t3)	−0.25 **	−0.36 ***	-						
		(t1/t2)	−0.21 **	−0.37 ***	0.78 ***						
		(t1/t3)	−0.18 *	−0.24 **	0.45 ***						
		(t2/t1)	−0.29 ***	−0.42 ***	-						
		(t2/t3)	−0.22 **	−0.30 ***	0.52 ***						
		(t3/t1)	−0.25 **	−0.23 **	-						
		(t3/t2)	−0.20 *	−0.35 ***	-						
4	Self-efficacy beliefs	(t1/t1)	−0.09	0.19 *	−0.01	-					
		(t2/t2)	0.05	0.28 ***	−0.09	-					
		(t3/t3)	0.05	0.15	0.15	-					
		(t1/t2)	0.02	0.38 ***	−0.09	0.79 ***					
		(t1/t3)	0.10	0.21 *	0.10	0.64 ***					
		(t2/t1)	−0.06	0.14	0.04	-					
		(t2/t3)	0.04	0.25 **	0.06	0.71 ***					
		(t3/t1)	−0.12	0.18 *	−0.02	-					
		(t3/t2)	0.00	0.20 *	−0.04	-					
5	Utility beliefs	(t1/t1)	−0.05	0.52 ***	−0.16 *	0.53 ***	-				
		(t2/t2)	−0.02	0.54 ***	−0.24 **	0.65 ***	-				
		(t3/t3)	0.20 *	0.58	−0.16	0.46 ***	-				
		(t1/t2)	−0.04	0.55 ***	−0.25 **	0.49 ***	0.80 ***				
		(t1/t3)	0.03	0.45	−0.05	0.43 ***	0.60 ***				
		(t2/t1)	−0.02	0.43 ***	−0.24 **	0.59 ***	-				
		(t2/t3)	0.14	0.40	−0.07	0.50 ***	0.59 ***				
		(t3/t1)	−0.06	0.32	−0.16	0.35 ***	-				
		(t3/t2)	0.05	0.37	−0.20	0.43 ***	-				
6	Reported SRL use (LIST)	(t1/t1)	−0.08	0.28 ***	−0.07	0.64 ***	0.61 ***	-			
		(t2/t2)	−0.06	0.22 **	−0.02	0.80 ***	0.60 ***	-			
		(t3/t3)	0.15	0.33 ***	−0.11	0.58 ***	0.57 ***	-			
		(t1/t2)	−0.01	0.30 ***	0.00	0.71 ***	0.60 ***	0.76 ***			
		(t1/t3)	0.05	0.33 ***	0.04	0.51 ***	0.58 ***	0.63 ***			
		(t2/t1)	−0.05	0.19 *	−0.03	0.59 ***	0.45 ***	-			
		(t2/t3)	0.06	0.22 ***	0.09	0.61 ***	0.48 ***	0.62 ***			
		(t3/t1)	0.01	0.19 ***	−0.11	0.49 ***	0.49 ***	-			
		(t3/t2)	0.03	0.21 ***	−0.11	0.52 ***	0.44 ***	-			
7	Reported metacognitive strategy use	(t1/t1)	0.26 **	0.16 *	−0.31 ***	0.08	0.05	0.00	-		
		(t2/t2)	0.25 ***	0.21 **	−0.32 ***	0.07	0.24 **	0.02	-		
		(t1/t2)	0.30 ***	0.20 *	−0.32 ***	0.08	0.12	0.02	0.69 ***		
		(t2/t1)	0.33 ***	0.20 *	−0.25 ***	0.04	0.13	0.01	-		
8	Reported cognitive strategy use	(t1/t1)	0.16 *	0.08	−0.24 **	0.04	−0.01	0.03	0.50 ***	-	
		(t2/t2)	0.20 *	0.16 *	−0.25 **	0.16 *	0.19 *	0.12	0.53 ***	-	
		(t1/t2)	0.23 **	0.12	−0.27 ***	0.10	0.07	0.04	0.39 ***	0.65 ***	
		(t2/t1)	0.25 **	0.14	−0.24 **	0.08	0.07	0.06	0.50 ***	-	
9	Problem-solving task performance	(t1/t1)	0.22 **	0.15	−0.34 ***	0.14	0.11	0.16	0.34 ***	0.47 ***	-
		(t2/t2)	0.21 **	0.29 ***	−0.35 ***	0.20 *	0.20 *	0.15	0.45 ***	0.56 ***	-
		(t1/t2)	0.08	0.22 **	−0.37 ***	0.18 *	0.15	0.12	0.31 ***	0.46 ***	0.57 ***
		(t2/t1)	0.27 ***	0.21 **	−0.30 ***	0.14	0.14	0.15	0.43 ***	0.38 ***	-

Note. SRL = self-regulation of learning, t1–3 = assessments before, directly after, and four weeks after the intervention. The first time point in the bracket refers to the point of time of the variable in the row, while the second refers to the point of time of the variable in the column. The variables metacognitive strategy use, cognitive strategy use, and problem-solving task performance have only been assessed at t1 and t2, hence no correlations for those variables for t3 are displayed. * *p* < 0.05, ** *p* < 0.01, *** *p* < 0.001.

**Table 5:** Results of multiple regression analyses.

Variable	*β*	*SE*	*z*	*p*	*R* ^2^
**Outcome variable: Reported SRL strategy use as assessed by questionnaire**					0.50
Consistent beliefs	−0.02	0.03	−0.47	0.64	
Inconsistent beliefs	−0.02	0.03	−0.58	0.56	
Self-efficacy beliefs	0.19	0.03	6.15	<0.001 ***	
Utility beliefs	0.18	0.04	4.88	<0.001 ***	
Self-efficacy × utility beliefs	0.02	0.03	0.79	0.43	
Knowledge	−0.01	0.03	−0.37	0.71	
**Outcome variable: Reported SRL strategy use as assessed in the problem-solving task**					0.14
Consistent beliefs	0.01	0.10	0.14	0.89	
Inconsistent beliefs	−0.22	0.09	−2.52	0.01 *	
Self-efficacy beliefs	0.00	0.09	0.01	0.99	
Utility beliefs	0.03	0.11	0.25	0.80	
Self-efficacy × utility beliefs	−0.17	0.08	−2.12	0.04 *	
Knowledge	0.13	0.08	1.69	0.09	
**Outcome variable: Performance on ADHD task**					0.27
Consistent beliefs	0.06	0.10	0.67	0.50	
Inconsistent beliefs	−0.22	0.08	−2.57	0.01 *	
Self-efficacy beliefs	0.20	0.10	2.05	0.04 *	
Utility beliefs	−0.04	0.11	−0.33	0.75	
Self-efficacy × utility beliefs	0.08	0.08	1.02	0.31	
Knowledge	0.17	0.08	2.29	0.02 *	
Reported strategy use (LIST)	0.02	0.11	0.14	0.89	
Reported strategy use (problem-solving task)	0.27	0.08	3.38	<0.001 **	
ADHD prior knowledge	0.00	0.08	−0.04	0.97	
**Outcome variable: Performance on giftedness task**					0.27
Consistent beliefs	0.17	0.09	1.86	0.06	
Inconsistent beliefs	−0.19	0.09	−2.18	0.03 *	
Self-efficacy beliefs	0.12	0.13	0.92	0.36	
Utility beliefs	−0.16	0.12	−1.35	0.18	
Self-efficacy × utility beliefs	−0.01	0.07	−0.09	0.93	
Knowledge	0.10	0.08	1.25	0.22	
Reported strategy use (LIST)	0.15	0.13	1.08	0.28	
Reported strategy use (problem-solving task)	0.32	0.08	4.04	<0.001 ***	
Giftedness prior knowledge	−0.02	0.07	−0.30	0.77	

Note. SRL = self-regulation of learning. * *p* < 0.05. ** *p* < 0.01. *** *p* < 0.001.

## Data Availability

The data presented in this study are available on request from the corresponding authors.

## Note

1 The study was originally planned so that the participants were randomly assigned to three groups, which would have resulted in a randomized experiment. However, at the time of data collection, in Germany, unpredictability and rapidly changing circumstances prevailed as to which kinds of data collections were allowed. We hence decided to randomly assign participants to two groups and once the threshold for two groups was reached, recruit the third group. For this reason, the design cannot be described as experimental, but only as quasi-experimental.

## Supplementary Materials

The following supporting information can be downloaded at: https://www.mdpi.com/article/10.3390/bs16040612/s1.
