# Supplementary material for: Misconceptions and Lack of Knowledge of Self-Regulation of Learning Hinder Students’ Use of Self-Regulation Strategies and Their Achievement: How This Can Be Changed by a Model-Based Instructional Video
Source: Behav Sci (Basel). 2026 Apr 20;16(4):612. doi: 10.3390/bs16040612 (PMC13113156; doi:10.3390/bs16040612)
Supplement: Supplementary file 1 [file behavsci-16-00612-s001.zip › Supplementary Materials S1.pdf]

Supplemental Material S1

The present Supplemental Material S1 displays the demographics of the three experimental conditions in the follow-up assessment.

Table S1

Demographics of the Three Experimental Conditions – Follow-Up Assessment

Variable	Mastery	Coping	Control	Total
n	51	46	43	140
Age	*M* = 23.20	*M* = 22.10	*M* = 22.30	*M* = 22.54
	SD = 3.70	*SD* = 2.70	*SD* = 3.66	*SD* = 3.40
Semester	*M* = 4.73	*M* = 4.74	*M* = 4.21	*M* = 4.57
	SD = 3.35	*SD* = 3.12	*SD* = 2.45	*SD* = 3.01
% female	80	87	71	84
% pre-service teachers	37	74	67	59
% psychology	12	15	5	11
% educational sciences	16	4	9	10
% sociology	35	4	7	16

Notes. Percentages of subjects do not round up to 100% due to NAs.
