# Supplementary material for: Misconceptions and Lack of Knowledge of Self-Regulation of Learning Hinder Students’ Use of Self-Regulation Strategies and Their Achievement: How This Can Be Changed by a Model-Based Instructional Video
Source: Behav Sci (Basel). 2026 Apr 20;16(4):612. doi: 10.3390/bs16040612 (PMC13113156; doi:10.3390/bs16040612)
Supplement: Supplementary file 1 [file behavsci-16-00612-s001.zip › Supplementary Materials S2.pdf]

# Supplemental Material S2

In the present Supplemental Material 2, we provide the materials that were presented in the two problem-solving tasks. We start by displaying the materials from the first task (ADHD) and continue with the second task (giftedness). For both tasks, we will show the order in which the materials were presented, the wording of the task itself, and the materials participants received. The instructional text was intentionally not placed at the top of the materials so that participants would have to get an overview of which materials they received in order to know what they had work on, i.e., so that they would exercise being self-regulated learners.

## ADHD Task

### *Order of Materials*

1. The book Gawrilow, C. (2023). *Lehrbuch ADHS: Modelle, Ursachen, Diagnose, Therapie*. UTB., on ADHD.
2. Instructional text
3. The (shortened) chapter "Role of the HAWIK-IV in the diagnosis of ADHD" from the book Petermann, F. & Daseking, M. (Eds.). (2009), *Fallbuch HAWIK IV*, Göttingen, Hogrefe.
4. The article „Wankerl, B., Hauser, J., Makulska-Gertruda, E., Reissmann, A., Sontag, T., Tucha, O. & Lange, K. W. (2014). Neurobiologische Grundlagen der Aufmerksamkeitsdefizit-/Hyperaktivitätsstörung. *Fortschritte Der Neurologie Psychiatrie*, *82*(01), 9–29. https://doi.org/10.1055/s-0033-1355710", which deals with the neurobiological basis of ADHD.
5. A shortened case study of a boy with ADHD symptoms named Paul from the book Petermann, F. & Daseking, M. (Eds.). (2009), *Fallbuch HAWIK IV*, Göttingen, Hogrefe.

6. Photographed book pages "ADHD - Diagnostic Criteria" from the Diagnostic and Statistical Manual of Mental Disorders (5th ed.; DSM–5; American Psychiatric Association, 2013).
7. The article „Visser, L., Büttner, G. & Hasselhorn, M. (2019). Komorbidität spezifischer Lernstörungen und psychischer Auffälligkeiten: ein Literaturüberblick. *Lernen und Lernstörungen*, *8*(1), 7–20. https://doi.org/10.1024/2235-0977/a000246", which deals with comorbidity of specific learning disabilities and mental health problems.

***Wording of the Task***

Dear participant,

Imagine you are a teacher. One of your main tasks as a teacher is to talk to parents in order to inform them about their children's problems at school. Imagine the following scenario: in 48 minutes, you will have a parent meeting in which you will have the task of informing Paul's parents about his behavioral problems. Please prepare notes in which you summarize all relevant information that you will address in the talk. Write down the notes in such a way that they could also be used by another person as a guide for the same talk (i.e. please be EXACT). At the beginning of the interview, you should briefly introduce Paul's parents to the causes and symptoms of ADHD. So that they understand thoroughly, you should also give at least one example per symptom of how the symptom generally manifests itself in the behavior of those affected. After that, turn to Paul's diagnosis and explain Paul's symptoms and the test profile. Please elaborate on which diagnostic criteria of ADHD Paul meets and what behavioral observations were made. Towards the end of the talk, review the implications of ADHD and why early intervention is important. Please note that the different options for intervention will be discussed in more detail in the second parent-teacher talk. Your current task is only to briefly point out different options.

## Giftedness Task Materials

### *Order of Materials*

1. The book „Ziegler, A. (2008). *Hochbegabung*. UTB GmbH.", which is a textbook on giftedness.
2. Instructional text
3. The article „Gnas, J., Müllensiefen, D., & Preckel, F. (2020). Was denken Musikschullehrkräfte über musikalisch hochbegabte Schülerinnen und Schüler? Eine experimentelle Untersuchung. *Beiträge Empirischer Musikpädagogik*, *11*, 1–33. Retrieved from https://www.b-em.info/index.php/ojs/article/view/182", which is an article on what music school teachers think about musically gifted students.
4. The article „Daseking, M., Petermann, F. & Waldmann, H. (2008). Der Allgemeine Fähigkeitsindex (AFI) – eine Alternative zum Gesamt-Intelligenzquotienten (G-IQ) des HAWIK-IV? *Diagnostica*, *54*(4), 211–220. https://doi.org/10.1026/0012-1924.54.4.211, which is an article on the General Ability Index (AFI).
5. The (shortened) chapter "Role of the HAWIK-IV in the diagnosis of the gifted" from the book Petermann, Franz & Daseking, Monika (Eds.). (2009), *Fallbuch HAWIK IV*, Göttingen, Hogrefe.
6. A shortened case study from a boy named Felix who might be gifted from the book Petermann, F. & Daseking, M. (Eds.). (2009), *Fallbuch HAWIK IV*, Göttingen, Hogrefe.
7. Photographed book pages, "Intellectual Giftedness" (p. 39-40) from the book Petermann, F. & Daseking, M. (Eds.). (2009), *Fallbuch HAWIK IV*, Göttingen, Hogrefe.
8. The article „Koop, Christine; Riefling, Markus: Schlussfolgerungen für die Weiterbildung von frühpädagogischen Fachkräften im Feld Hochbegabung - In: Koop,

C. & Riefling, M. (Eds.): Alles eine Frage der Haltung!? Begabtenförderung in der Kindertagesstätte. Frankfurt : Karg-Stiftung 2017, S. 67-73. - (Karg Hefte. Beiträge zur Begabtenförderung und Begabungsforschung; 10) - URN: urn:nbn:de:0111-pedocs-140518 - DOI: 10.25656/01:1405", which is on conclusions for the further training of early childhood professionals in the field of giftedness.

***Wording of the Task***

Dear participant,

Imagine you are a teacher. One of your main tasks as a teacher is to talk to parents in order to inform them about their childrens' problems in the school context. Please imagine the following scenario: In 48 minutes, you will have a parent meeting in which you will have the task of informing Felix's parents about his possible giftedness. In preparation for the talk, please prepare notes in which you summarize all relevant information that you will address during the talk. Please write down the notes in such a way that they can also be used by another person as a guide for the talk (i.e., please be EXACT).

At the beginning of the interview, you should briefly introduce the parents to the traditional definition of giftedness, explain what is meant by underachievement, and what the potential causes of giftedness are. Then you turn to Felix's diagnosis and first address the current problem and peculiarities. In addition, you should discuss the intelligence profile, the overall result, and also sub-tests, strengths and weaknesses, and what behavioral observations were made during the testing. Based on this, you will address to what extent the diagnostic criteria are met, and based on which values you come to your conclusions. At the end of the conversation, it is important to point out opportunities for support and the general effects that can be expected from such support measures.
