# Supplementary material for: Misconceptions and Lack of Knowledge of Self-Regulation of Learning Hinder Students’ Use of Self-Regulation Strategies and Their Achievement: How This Can Be Changed by a Model-Based Instructional Video
Source: Behav Sci (Basel). 2026 Apr 20;16(4):612. doi: 10.3390/bs16040612 (PMC13113156; doi:10.3390/bs16040612)
Supplement: Supplementary file 1 [file behavsci-16-00612-s001.zip › Supplementary Materials S3.pdf]

Supplemental Material S3

Supplemental Material S3 shows the coding schemes for the ADHD (Table SS) and giftedness (Table S3) task.

Table S2

ADHD Task Coding Scheme

	Criteria	Sample Solution	Rating
1	**Introduction to ADHD**:		
	Causes	- Genetic factors<br>- Structural and functional deviations in the brain/ neuropsychological factors<br>- Psychosocial factors<br>- Environmental influences<br>- Adverse family circumstances<br>- Monoamine-Deficiency Hypothesis	- 1P. – at least 1 cause mentioned<br>- 2P. – at least 2 causes mentioned<br>- 3P. – at least 3 causes mentioned
	Symptoms	- Inattention (e.g., careless mistakes, distractible, forgetful, difficult to maintain attention during tasks)<br>- Hyperactivity (e.g., fidgeting, excessive talking, plays restlessly, difficulty speaking calmly)<br>- Impulsivity (e.g., difficult to wait, interrupting others, blurting out answers)	- 1 P. – 1 symptom plus example mentioned<br>- 2 P. – 2 of the core symptoms incl. examples mentioned<br>- 3 P. – all 3 core symptoms incl. example mentioned
2	- **Paul's diagnosis:**		
	Symptoms and behavioral observation	Symptoms:<br>- Easily distracted<br>- Loud during play<br>- Increased urge to move<br>- Does not complete written work independently<br>- Motoric restlessness + impulsive behavior<br>- Fights with other children<br>- Temper tantrums<br><br>Behavioral observation:<br>- Distracts himself by thinking out loud<br>- Needs breaks and time to concentrate<br>- Distractible, but goal-oriented<br>- Cannot complete written work on his own<br>- Slowness<br>- Clear signs of fatigue<br>- Large vocabulary<br>- Humorous<br>- Very cooperative<br>- No insecurities/ fears	- 1 P. – 1-2 symptoms mentioned<br>- 2 P. – 3-4 symptoms mentioned<br>- 3 P. – at least 5 symptoms mentioned

	Criteria	- Sample Solution	- Rating
	Test profile (HAWIK-IV)	- Average overall score/ intelligence (in language comprehension, perceptual reasoning, working memory)<br>- Below average in processing speed → often goes along with ADHD	- 1 P. – HAWIK only mentioned<br>- 2 P. – HAWIK result correctly named<br>- 3 P. – HAWIK result plus reference to ADHD
	Which diagnostic criteria of ADHD does he meet?	(either written down or marked in notes)<br>- Inattention<br>- Hyperactivity<br>- Impulsivity	- 1 P. – 1 criterion<br>- 2 P. - 2 criteria<br>- 3 P. – at least 3 criteria
3	- **Consequences of ADHD:**		
	Reasons for early intervention/ importance	- Limitation in school and performance<br>- Increased risk behavior, risk of depression and risk of self-medication in adolescence<br>- Lifelong disorder<br>- In adulthood: more work-related difficulties, deficits in social skills<br>- Without diagnosis and intervention: affected people blame themselves for difficulties (anxiety, depression, etc.)	- 0P. – only urgency emphasized without justification<br>- 1P. – at least 1 reason named<br>- 2P. – at least 2 reasons named
	Options for intervention	- Behavioral interventions in school (e.g., choices, praise, punishment)<br>- Self-regulation, self-management (e.g., find own learning strategy)<br>- Training programs<br>- Drug therapy (e.g., MPH, Ritalin)<br>- Structuring measures in everyday life (e.g., daily routine, calendar, workplace)<br>- Sport and nature<br>- Praise<br>- Self-help groups for parents<br>- Collaboration with teachers	- 1P. – 1 intervention named<br>- 2P. – 2-3 interventions named<br>- 3P. – at least 4 interventions named
			Max. 20 points

Table S3

Giftedness Task Coding Scheme

	Criteria	Sample Solution	Rating
**1**	**Introduction to giftedness**		
	Traditional definition of giftedness	- IQ min. 2 SD higher than mean (100)<br>- IQ higher/ minimum 130/ percentage range > 98, T-value > 70<br>- Definition of IQ is necessary, but not sufficient/ is being criticized	- 1P. – at least 1,<br>- 2P. – at least 2,<br>- 3P. – at least 3 of the bullet points on the left
	Under-achievement	Underachievement:<br>- Talent whose performance is currently impaired, no intervention results in unfavorable prognosis for the achievement of excellence performance<br>- Discrepancy between intelligence and (school) performance<br><br>Potential factors that lead to an unfavorable performance development:<br>- Insufficient motivation<br>- Inadequate learning and working behavior<br>- Motoric deficits<br>- Personality psychological parameters (attribution styles, success expectations and personal values)<br>- Environmental factors (beliefs in gender roles, stereotypical jobs<br>- Lack of support and insufficient learning resources	- 1P. – „Definition"<br>- 2P. – „Definition" + 2-3 reasons/ factors<br>- 3 P. – „Definition " + min. 4 reasons/ factors
**2**	**Diagnosis of Felix**		
	Charac-teristics, current problem	- Denial of performance in school, getting bad grades<br>- Risk of moving up<br>- Parents interpret the problem as a motivational one<br>- Teachers see him as mentally absent, rarely participates<br>- Does not like to go to school, describes himself as internally restless, easily distracted by external stimuli<br>- Dalliance, negligence<br>- Parents underline great willingness to help, inventiveness und imaginative playing<br>- No peculiarity during school or Kindergarten (besides some violations of rules, no anomalies)	- 1P. – at least 2,<br>- 2P. – at least 3,<br>- 3P. – points at least 5 of the bullet points on the left
	Description of HAWIK-profile	- Above average total result<br>- IQ = 119<br>- Heterogeneous profile, 31 IQ points between the highest and lowest index<br>- Verbal Comprehension Index (VCI) and the Perceptual Reasoning Index (PRI) above average<br>- GAI and Cognitive Proficiency Index (CPI) average range<br>- Pairwise discrepancy analysis: all Index pairings, which includes the GAI and CPI indices are clinical relevant<br>- Key performances: Mosaic-test, vocabulary-test<br>- Weaknesses: to repeat numbers, number-symbol test	- 1P. – above average total result, IQ = 119<br>- 2P. – additionally mention the heterogeneous profile, VCI, PRI above average and GAI and CPI average range<br>- 3P. – additionally name pairwise discrepancy analysis / Key performances and weakness

	Criteria	Sample Solution	Rating
	Fulfillment of criteria/ GAI	- Interpretation of the total-IQ regarding heterogeneous profile, is only possible to a limited extent, therefore General Ability Index (GAI) should be used<br>- GAI = 128<br>- Results have to be assigned to giftedness (referring to GAI)<br>- High expression of differences between index pairings of VCI/ PRI and GAI/ CPI which are typical for higher performance by children and youth	- 1P. – fall back on General Ability Index<br>- 2P. – interpretation of index or mentioning Index pairing
	Behavioral observation	- No anomalies<br>- Resilient<br>- Open minded und highly motivated<br>- Well concentrated<br>- No impairments by external stimuli<br>- Conspicuous: verbal articulacy<br>- Good management of subjectively experienced failure<br>- Work on all exercise	- 1P. – at least 2,<br>- 2P. – at least 4 of the bullet points on the left
**3**	**Consequences of giftedness**		
	Possibilities of support	- Acceleration (going through a curriculum faster, e.g., early enrollment in school, skipping grades, temporary participation in lessons of higher grades, individualized learning with self-determined speed in learning, tightening and shortening the learning plan, courses during vacation, high-speed classes)<br>- Enrichment (enrichment of regular curriculums, e.g., enroll for additional courses, field trips, join higher classes, extracurricular events, study-group, summer courses, extracurricular private lessons)<br>- Pull-out program (weekly talent development programs)<br>- Performance based groups (homogenous leaning groups regarding their performance)<br>- Intervention-oriented support approach (depending on the goal of the intervention setting fall back on successful methods)<br>- Eclectic support approach (identify successful support measures, and use these as parts for a general support of giftedness)<br>- Support approach of pursuing excellence (curriculums are oriented to achieve a high level on performance)	- 1P. – at least 1 named or described roughly<br>- 2P. – at least 2 named or described roughly<br>- 3P. – at least 3 named or described roughly
	General Effectiveness	Which effects of support measures are possible?<br>- Until now, effects of support measures are low to moderate<br>- One standard deviation of effect for known support measures<br>- Improvement of 0,6 grade points at most	- 1P. – at least 1 named
			Max. 20 points
