# Supplementary material for: Misconceptions and Lack of Knowledge of Self-Regulation of Learning Hinder Students’ Use of Self-Regulation Strategies and Their Achievement: How This Can Be Changed by a Model-Based Instructional Video
Source: Behav Sci (Basel). 2026 Apr 20;16(4):612. doi: 10.3390/bs16040612 (PMC13113156; doi:10.3390/bs16040612)
Supplement: Supplementary file 1 [file behavsci-16-00612-s001.zip › Supplementary Materials S4.pdf]

Supplemental Material S4

In the present Supplemental Material S4, one can find the example we used to denmonstrate how participants could describe what they were doing in retrospect in the problem-solving tasks.

Example for the Description of One's Procedure

Imagine you are cooking some spaghetti with tomato sauce for lunch. In between, your roommate, friend, or similar person asks you what you have been up to in the last ten minutes. Your answer could be, for example:

"First, I picked out a recipe for the tomato sauce. To do this, I browsed through all the cookbooks in our apartment. Actually, I know how to cook tomato sauce, but I wanted to cook a larger portion and was unsure of the quantities of ingredients I would need. I skimmed a few recipes and first ruled out all the ones for which I didn't have all the ingredients available. Then I settled for one for which I had all the ingredients available.

Then I wrote down all the ingredients I needed for the sauce, gathered them, and laid them out on the counter. I also considered all of the amounts that were in the recipe in grams. I almost measured out too much salt, but luckily I saw that the recipe required a smaller amount.

Then I worked through the individual steps of the recipe: I first heated olive oil in a pot and finely chopped and fried onions. Although, first, I had to sort out some onions that were a bit mushy. At the same time, I put on some water to boil. Now I'm just waiting for that and preparing the next ingredients for the sauce."

This is an example of what describing your own procedure might look like.

In the following task, please describe your approach to the task in this same style and level of detail.
