# Supplementary material for: Misconceptions and Lack of Knowledge of Self-Regulation of Learning Hinder Students’ Use of Self-Regulation Strategies and Their Achievement: How This Can Be Changed by a Model-Based Instructional Video
Source: Behav Sci (Basel). 2026 Apr 20;16(4):612. doi: 10.3390/bs16040612 (PMC13113156; doi:10.3390/bs16040612)
Supplement: Supplementary file 1 [file behavsci-16-00612-s001.zip › Supplementary Materials S5.pdf]

Supplemental Material S5

Supplemental Material 5 displays the video script for the mastery and coping modeling, as well as for the control group video.

Mastery Modeling Video Script

Hi guys! As announced yesterday, today I want to show you how I proceed when I work on exercises for my studies. For all of you who don't know me yet: I'm Anna and I'm studying to be a teacher in my fifth semester. For my studies I had to hand in assignments from time to time and so far I have always received very good feedback.

In the first semesters of my studies, I didn't really know how to approach such assignments, which is why I attended a workshop on self-regulation of learning offered by the university. What I learned there translates easily to working on assignments for university.

In the workshop, I learned that there are different strategies that can be used to be structured and self-regulated when working on assignments. In the workshop the leaders said that you can learn very well how to use these learning strategies successfully and scientific findings have also shown that students who use such learning strategies perform better. So today I want to show you how I use my knowledge from the workshop when working on assignments for university. Have fun watching.

Because of the workshop on learning strategies, I know that you can use helpful strategies even before you really get started with the actual work. One of these helpful strategies is to get an overview of the assignment and the materials provided so that you know what to pay special attention to when working on the assignment. For example, it's important to think very carefully about what you're supposed to be doing. Then you need an overview of the texts and information that you have and that you still need to find. Then you can plan the exact procedure. Such a plan helps you to keep track of the individual steps to complete the exercise and your own time management and to organize yourself. For good planning, for

example, you make a schedule and think about when you want to work on what. In terms of content, you think about which texts and tasks you want to start with and in which order you want to read the texts and work on the task. For me, this means that I usually first have a look at what requirements my seminar instructors have for an assignment - i.e. what scope, what topic should be worked on, by when the exercise should be handed in, and so on. Today, for example, I saw that the assignment was to develop a ten-page lesson plan for a ninth or tenth grade class on a topic of my choice, incorporating digital media to support it. Then I looked to see what materials I already had on the topic, for example, information from lectures and seminars and literature references that the seminar instructor had provided. I then put these texts at hand and skimmed them to get a first overview of the topic of each text. After that, I had a better impression of how much time I would have to invest in each step of the assignment and which texts I would really need for this assignment and which ones I would not.

Afterwards, I always write a concrete plan for myself, which task I want to have completed and when. I also think about by when I want to be finished, what materials I need for what, and so on. While I'm writing the plan, I'm also setting goals that are as specific as possible. Setting goals helps me a lot to be more motivated to work.

Today, I set the goal that I want to finish the assignment onne day before the submission date so that I can go through it again at my leisure. I also thought about in which subject and with which class I would plan the lesson and what the topic and learning objective of the lesson should be. I have already briefly considered how digital media can be used in the process.

Then I prepare additional materials that I need for the task. I do this by researching online and borrowing suitable books from the literature list. This precautionary preparation of

all required materials helps me to work in a concentrated manner and without unnecessary interruptions that would result from a later search for materials.

Afterwards, I started to implement my plan and, for example, read through selected texts and made notes on the individual lesson sequences and then also started to work out the text. I also marked the most important points in the texts that I didn't understand right away. That way I knew what was relevant to the topic and understood them better. Some of my fellow students think that if you don't understand a text the first time, you won't understand it the next time you read it, but in my workshop on self-regulation of learning I learned that this is not true and that you can very well do something to understand texts better. Also, some are convinced that successful students should be able to learn something quickly, but again, I realized that you can be a successful student even if you need a little more time to understand some topics. In the workshop I also learned that it is helpful to use strategies even while working. The most important thing is to monitor yourself while working and whether you are really doing what is conducive to the task processing, what brings you closer to achieving your goals and what is favorable for your own time management, or whether you are just doing something else. So I kind of observe myself on a meta-level while I'm working. That always helps me stay on task and stick closely to my plan. If I drift off, I notice it directly and can take countermeasures. I know from my previous studies that it is a weakness of mine that I get distracted quickly. Knowing my strengths and weaknesses in learning also helps me a lot to recognize when I should invest more time or ask others for support.

At regular intervals, I also look to see whether I have achieved my goals and then check these goals off in my schedule. I also reflect on how satisfied I am with my progress so far. Finally, it is also important to use strategies after the actual learning and working phase. This reflection helps me to see if there is anything I would like to change in my approach that will then better help me achieve my goals. Today, for example, I scheduled more time for

reading into how to create lesson outlines because I still encountered a few gaps - but such adjustments to the plan are also part of self-regulation of learning.

Also, while reflecting, I realized that I found the use of learning strategies super useful today and they are really worthwhile for studying to make better progress. I also realized that I find it totally useful when teachers not only pass on content knowledge to their students, but also teach them which strategies they can use to learn successfully. When I finished the exercise, I decided to think about how I could do even better on the next task. This is also part of self-regulation of learning.

If you want to try self-regulation of learning yourself, here is a summary of the steps: Self-regulation in learning includes getting an overview, making a plan and setting goals and picking out materials and monitoring yourself during the work process and keeping track of your own time management, as well as checking to see if you've achieved your goals and reflecting at the end of the day to see if maybe you could do something better next time. You can use these steps, as I did today, when working on exercises, but also when writing essays, preparing for presentations or studying for exams. By the way, you don't only need these learning strategies, as many people think, when you encounter difficulties, but they help in almost all learning situations, as I showed you today. This way you end up working more effectively and also saving a lot of time.

I hope you enjoyed the video. Thank you so much for watching today!

Coping Modeling Video Script

Hey guys! As I announced yesterday, I'll show you today how I worked on my first exercise for my studies. For all those who don't know me yet: I'm Anna and I'm studying to be a teacher in my first semester. For a seminar I have to hand in an assignment for the first time. Actually, I wanted to upload a video yesterday, but I didn't make any progress at all. Today I

tried something else and I'm much happier with the result. That's why I'll show you today how I worked yesterday and today and how it worked out. I hope you have fun watching!

First of all, let me show you what I did yesterday. My task was to design a lesson plan and to integrate digital media in a meaningful way. I was able to choose the topic of the lesson myself. In order to get the task over with quickly, yesterday I started with googling how to use digital media in the classroom. I didn't feel like wasting time on considering in advance exactly how to proceed. Some people get everything ready first, but that takes too much time and is not worth it. When googling, there immediately were a lot of hits and I then started to read the hits in detail and dealt intensively with the topic. I did not understand some of the texts immediately. I didn't look at them any further, because I don`t think I would understand them if I read them several times. It's really a waste of time to try to understand something that didn't make sense the first time I read it. At some point I noticed how time flies. By that time, I had been busy for quite a long time. After that, I did have some ideas about how to use digital media in the classroom. Unfortunately, it was very difficult for me to decide on a teaching topic because there are so many possibilities. I then came across some suitable and many not suitable Youtube videos. Some were also really informative, only unfortunately I was then distracted for a while by the videos in the queue. Since I still hadn't decided on a topic at that point, I then without further ado chose the last topic I had watched a video on due to time constraints. After that, I started working on the actual lesson outline. I tried to convey a lot of subject knowledge to the students in the lesson. After all, that's my job as a teacher. I worked on that for a while first. After a while, I noticed that I hadn't really read through the exact assignment. I made up for it and realized that my draft was much too short. My seminar leader wants ten pages and I had only written five, bummer! Actually, I thought I could still remember the assignment well from the seminar. Besides, the lesson was supposed to be designed for a completely different grade level than I had thought about. Therefore, both my topic and the digital media I wanted to use were not very fitting. This then really frustrated me

as I realized that I could almost start all over again. I also doubted whether studying really suited me, as other students would certainly complete this task much faster. After all, if you're good, you're also fast. I thought to myself, if it takes me so long to do the task, maybe I'm not cut out for studying. At some point, a friend called me and asked me if I had time to talk on the phone. At that point, I didn't feel like working anymore anyway and turned off the laptop. I told my friend that I was really unhappy with my day and that I had to change something if I wanted it to work better the next day, but I was unsure exactly what I could do differently. She then said that she had recently attended a workshop on self-regulation of learning. She told me about many learning strategies and explained how I can also proceed in a structured and self-regulated way when working on an exercise. At first I was very skeptical and thought it was a total waste of time. You can't really learn how to successfully work on such a task. Either you can do it, or you can't. Some people are just better at it than others. But my friend really recommended the strategies to me and I tried to implement them the next day. It took a bit more time at first, but actually I would have been faster if I had already taken the time yesterday. You can actually learn learning strategies and it's worth it, even if I wouldn't have thought that before.

After talking with my friend about learning strategies, I understood that you can use these helpful strategies even before you start with the actual work. I wouldn't have thought that at all. I used to jump right into the task, thinking I would finish faster that way. One of these helpful strategies is to get an overview of the assignment and the material provided so that you know what you should pay special attention to while working on the assignment. For example, it is important to think very carefully about what you are supposed to do. To do this, it helps to read the assignment really well so that you don't overlook anything and work in a completely wrong direction like I did yesterday. Then you need an overview of the texts, the information that you have and that you still have to find out. Then you can plan the exact procedure. Such planning helps to have the single steps to work on the exercise and the own

time management in mind and to organize oneself and prevents that one starts completely aimlessly with something and then notices that it was not at all purposeful or helpful like I did yesterday. For good planning, you make a time plan and think about when you want to work on what and in terms of content you think about which texts and tasks you want to start with and in which order you want to read them and work on the tasks. So this morning I first had another look at exactly what my seminar instructor's assignment was - i.e. what scope, what topic is to be worked on, by when the exercise assignment is to be handed in, and so on. If I had done this yesterday, I would have seen directly that I was supposed to develop a ten-page lesson plan for a ninth or tenth grade level on a topic of my choice, incorporating digital media to support it. Unfortunately, I didn't have that information yesterday. Then I looked at what materials I already had on the topic, e.g. information from lectures and seminars and literature references provided by the seminar instructor. So I wouldn`t have needed to google yesterday. Today, I had all of these texts ready and skimmed them to get a first overview of the topic of each text. After that I had a much better impression of how much time I still have to invest in each step of the task and which texts I really need for this task and which I don`t need. I also realized that yesterday I really wasted time on texts and videos that I didn't need in the end.

Afterwards, I wrote down a tangible plan of what tasks I wanted to have completed for the exercise and when. I also thought about by when I would like to be finished, what materials I need for what and so on.

While I was writing the plan, I also set myself tangible goals, because my friend also gave me this tip. For her, goals help a lot to work in a more motivated way. So I set myself the goal that I would like to have finished the exercise one day before the deadline, so that I can go through it again at my leisure. Then I thought about in which subject and with which class I was planning the lesson and what the topic and learning objective of the lesson should

be. I have already briefly thought about how digital media can be used here. If I had done this yesterday, I wouldn't have wasted my whole afternoon.

Afterwards, I prepared additional materials that I would need for the task. I researched online - but this time not as chaotically as yesterday - and borrowed books from the library. According to my friend, this precautionary preparation of all needed materials can help to work concentrated and without unnecessary interruptions that would result from a later search for materials. Unlike yesterday, I actually felt like I had prepared well and started with a good plan and a goal in mind and I didn't get distracted from working by YouTube videos like I did yesterday.

Whilst reading, I remembered that my friend also advised me to use strategies even while working. The most important thing is to monitor myself while working, whether I am really doing what is conducive to the task processing, what brings me closer to achieving my goals and what is favorable for my time management, or whether I am just doing something else. So I then sort of monitored myself working on a meta level. Yesterday I didn't manage to do that and I always got distracted quickly, which is probably a weakness of mine. Getting to know my weaknesses and strengths will also help me in the future to recognize when I need to invest more time or ask others for support.

Monitoring worked so well and I was able to work with such concentration that I also wanted to follow my friend's last tips. Namely, it's apparently important to use strategies even after the actual learning and working phase, and to regularly reflect on whether you've achieved your goals and consider how satisfied you are with your progress so far. This reflection probably helps you to see if there is anything you would like to change in your approach that would then better help you to achieve your goals. Actually, at first I found it an annoying idea to think about it further after completing the task. However, my friend convinced me that the extra effort, which was then quite short, was worth it. I then checked

off the goals I had achieved in my plan. In the process, I noticed that I had not achieved all of my goals, so I slightly adjusted my plan again. According to my friend, such adjustments to the study plan are also part of self-regulation of learning. Today, I scheduled more time to read into creating lesson outlines because I still came across a few gaps. Also, while reflecting, I realized that my own attitude towards learning strategies has changed because their use worked so well for me today. I now believe that it is really worthwhile to use learning strategies for studying in order to make better progress and actually I think it is really important that teachers do not just pass on subject knowledge, but also teach their students what strategies they can use to learn successfully.

When I finish the practice task, I set out to figure out how to do even better on the next task. This is also part of self-regulation of learning.

Overall, I was much happier with the result today than yesterday and I want to continue working like this tomorrow. I also realized today that, contrary to what I thought, successful learning doesn't always happen quickly, and sometimes you have to invest more time to understand something correctly. In the end you can still be successful. If you want to try self-regulation of learning, here is a summary of the steps: Self-regulation of learning involves getting an overview, then making a plan, setting goals, and picking out materials, and monitoring yourself during the work process and keeping track of your own time management. It also involves checking to see if you have achieved your goals as well as reflecting at the end of the day to see if there is something you can do better next time. You can use these steps, as I did today, when working on exercises, but also when writing homework, preparing for papers or studying for exams. By the way, you don't only need these learning strategies when you encounter difficulties - like I did yesterday - but you can always apply them and in the end you will work more effectively and also save time.

I hope you enjoyed the video. Thank you very much for watching today!

Control Group Video Script

Sunday evening: Hey guys, welcome to a new video. For those of you who don't know me yet: I'm Anna, and I'm studying to be a teacher, and I'm in for a very exciting time, because my block internship is about to start. That means that starting tomorrow, I'll be at an elementary school for four weeks. That will be a very exciting time. It looks like I'm going into a fourth grade class and I was already there last week with my mentor and got to know all the kids and it was really sweet. They all seem really kind. Since I have a fourth grade class, I think I always have five or six school hours, that is, lessons, in which I can sit in. Actually, I wanted to get ready to go to bed because I want to be well rested for tomorrow, but I thought I'd tell you a little bit about how I'm feeling before my first day. I also had to think about a main topic for my portfolio, which I have to write afterwards. That's really great, I've already talked to my mentor about it and it looks like I'll be writing about classroom disruptions. That's also very practical, there's already so much literature on it. I don't think I can do much wrong. Unfortunately, I can't film in class – because of privacy reasons and so on - so I can't share the kids and the actual impressions with you, but I'll report every evening how it went. To be honest, I'm really looking forward to looking at it again in a few years or a few months and maybe having a completely different view of my experience. So I'm looking forward to reminiscing about the videos at some point and hope you enjoy it. I hope it helps you. Now I'm going to get ready for bed and go to sleep as soon as possible, so that I'll be well rested tomorrow.

Monday: Hi guys! I'm reporting back. I had my very first day of my block internship today and if I'm honest, I'm pretty beat. It was super exhausting, so many new impressions, but my class is also really, really, really loud. So, it's going to be a challenge. But it was still fun, especially when we had Physical Education in the fifth and sixth hour. I had two English lessons first, then Maths, and then P.E. last. It was nice, because then we could really get the kids to work off some energy. I think it was really good for them and I had the feeling that

they were in a better mood afterwards and were a bit calmer. I'm curious to see if that's always the case. But what was really sweet was that we had a morning circle this morning and all the kids talked about their weekend. One child told us that it was her birthday and everyone started singing birthday songs. They all didn't want to stop and it was really sweet and I thought "Okay this is going to be a good time". What else… In English we did vocabulary, so we did some revision work. I didn't really know what to do then. I had the feeling that if I walked through the aisles with the kids and looked over their shoulders, they would be totally distracted again. That means I actually let them go through the vocabulary by themselves for maybe 20-30 minutes and held back. Tomorrow is a day at my old elementary school, because my mentor also works at that elementary school, where I used to go to school myself as a child. I'm really looking forward to seeing how it's changed, and maybe I'll even get to see my own teacher from back then. That would be really nice. I'm definitely excited and I'll keep you posted.

Tuesday: Hi! Today was my second day of my block internship and I was at my old elementary school and that was really really nice. I first had a fourth grade class and we gave English lessons. It was really sweet, the kids were really nice and calm and it was fun. What was really nice was that I was also in a third grade music class and was allowed to sit in. The kids were so curious, it was really nice to see. They took all the instruments into their hands and played wildly on them, but also listened when you wanted to explain something to them. It was really sweet and nice that they were so fascinated by the musical instruments. Then, of course, we sang lots of songs together and that was really fun. After that - after the sixth lesson - I went to the teachers' room and looked around a bit to see what had changed there and then I actually met my old teacher, whom I hoped to see as I said yesterday. I think I haven’t seen her for 20 years. That was really crazy. She had a problem with the printer and I helped her out. I knew this printer from an old job, so I found it interesting that all the job experiences that you have in your life and that you make can help you in the end in other

situations. That was really nice and I was able to connect a bit with the teachers there and talk to them. That was a good chance. Tomorrow I'm going back to the first school, so I'm looking forward to that.

Wednesday: Hi! Today was my third day at school and I honestly don't have that much to tell. My day was pretty short. I was only at school for four hours and I don't have much of an update. In the first two hours the kids had Maths and I think that was quite a challenge for them. They struggled quite a bit. Then we had German. What's actually interesting to tell is that today I supported a couple of teachers in the break time supervision. There was even a small conflict, nothing big. But I had to intervene, some students were playing soccer in an area where they are not allowed to play soccer. There are separate areas for ball games. That's why I went there and explained to them that they weren't allowed to do that there. They were so nice, I was really surprised. They apologized right away and said that they wouldn't do it again and that they were sorry. They immediately went to another area, so that the other students who were playing other things could continue in a relaxed way. It was very nice to see that actually nobody was alone in the schoolyard. I had the feeling that everyone was actually in groups and everyone felt well taken care of. I think tomorrow I will be able to tell you a bit more about the lessons. I think there will be more things happening. But for today, that's it for now.

Thursday: Hi. I just got home from school and today was a little more exciting than yesterday. I had six lessons and four of them I was with my internship class. That was really nice and really fun and also went better than the first day. After that I had another special tuition lesson. But there I mainly observed. There weren't many students there at all. I just watched that for the time being. Then I had German as a second language as my very last lesson. There, too, I primarily just watched and helped with questions. It was very impressive to observe how quickly small children can grasp languages, so it was really amazing. That was really nice to see. Just now I received the timetable of the fourth grade from my old

elementary school, where I had been before. Now I have to take another look at it and see how I'm going to manage with all the courses I still have to take. That's all there is today. See you tomorrow.

Friday: Hi, I just got out of school and yes now my first week of teaching at the elementary school is already over and the first week of the block internship. I must say I am a bit exhausted. I had six lessons in my internship class and then an extra lesson, in which I sat down with my mentor and we talked about how I prepare for the lessons that I will give myself. We said that we want to approach it a bit slowly at first, that in the next few weeks or primarily in the next week I first always take over the first 15 minutes of lessons and thus then prepare for the lessons at the end, where I then have to hold classes for 90 minutes. So I was already thinking that I would like to do that in English. I think the topic “hobbies” would be nice. I think that would be fun for the kids. But first I have to prepare 15 minutes of the Maths lesson on Monday. The kids are also writing a Maths exam next week and I'm now responsible for preparing an exercise in which they prepare well for multiplication and recall everything back to memory. For the weekend, I get to work on preparing well for it and to think of great things. I have a few ideas already. But yes, that will be the focus of the weekend now. I can't believe that the first week of my block internship is already over. It went so fast and I experienced so many different things and at the same time it felt really long. I hope the first week was a bit informative for you and maybe also fun. I had a lot of impressions and I think there will be a lot more to come. I am in any case curious and will keep you up to date. Now I wish you first of all a very nice weekend and yes I'm looking forward to a bit of relaxation. Until next week.
