# Supplementary material for: Misconceptions and Lack of Knowledge of Self-Regulation of Learning Hinder Students’ Use of Self-Regulation Strategies and Their Achievement: How This Can Be Changed by a Model-Based Instructional Video
Source: Behav Sci (Basel). 2026 Apr 20;16(4):612. doi: 10.3390/bs16040612 (PMC13113156; doi:10.3390/bs16040612)
Supplement: Supplementary file 1 [file behavsci-16-00612-s001.zip › Supplementary Materials S6.pdf]

Supplemental Material S6

Supplemental Material S6 displays the changes to the coding scheme used by Greene and Azevedo (2009). We made those changes because (a) either the codes that were used by the authors were developed specifically for a hypermedia-learning environment and did not fit our data, (b) because we could not distinguish between codes in our retrospective reflection data or (c) because we added codes that we found in our data which were not yet reflected in the coding scheme by Greene and Azevedo (2009).

Table S4

Retrospective Reflection Coding Scheme Based on Greene and Azevedo (2009)

Code	Description	Examples	Changes to G&A
0) No Strategy	For cases where none of the following codes fit		Code added
**MACRO-LEVEL PROCESS: PLANNING**			
1) Orientation/ Planning	Orientation:<br>- Happens on the one hand before planning - e.g., you get an overview of the materials.<br>- Also when skimming text contents (then also code code 13 - reading).<br>- On the other hand, e.g., the creation of an outline/ guide always serves as orientation.<br>Planning:<br>- A plan involves coordinating the selection of operations; execution requires behavior oriented to the problem and goals / subgoals.<br>→ This is about different steps of action<br>→ planned action (simply "working through" something, "dealing with" something) is not sufficient<br>→ "first X, then Y..."	"I first tried to get an overview of the task materials."<br><br>"First I will look at the environment, then I will go to the specific sections of the circular system"	Orientation added as a sub-code and merged with Planning

Code	Description	Examples	Changes to G&A
2) Goals	Goals include operations that are possible, postponed, or intended, or states that are expected to be achieved. Goals can be identified because they do not refer to states that already exist<br>→ they are specifically about what one wants to achieve through the planned action steps<br>→ "to" often used as indicator for goals	Learner looks at learning environment, for example - "... to understand its structure."	
3) Prior knowledge activation	Search in memory for existing knowledge related to the topic - before or during task execution.<br>→ if necessary, code together with 26 - Knowledge elaboration	"It's difficult to understand, but I vaguely remember learning about the role of blood in high school."	
4) Recycle goal in working memory	Repeat the goal (e.g., question or parts of a question) in working memory.<br>Memorize goal in working memory (e.g., question or parts of a question)<br>→ Rather not code together with 2 - Goals.	"...describe the location and function of the main valves in the heart."	
**MACRO-LEVEL PROCESS: MONITORING**			
5) Judgment of learning/ Judgment of knowing/ Meta-cognitive awareness	Judgment of learning: Learner becomes aware of (not) understanding what is read - relates more to content learning.<br>Judgment of knowing: Learner becomes aware that he does not know something/ has no prior knowledge about something.<br>Metacognitive awareness: Learner is aware of his learning process in general.	"I don't know this stuff, it's hard for me.";<br>"Determined that I don't know anything about ADHD."	Extended by Judgement of knowing and Metacognitive Awareness
6) Feeling of knowing	Learner is aware of having read and understood something in the past; but cannot recall and reproduce this on demand - relates more to existing knowledge	"Let me read this again until I begin to understand it."	
7) Self-questioning	Asking yourself a question and rereading if necessary to increase understanding<br>Thinking about the task at hand	The learner reads a text, then asks him/herself "What do I know about this?" and reviews the same content if necessary	

Code	Description	Examples	Changes to G&A
8) Content evaluation/ Evaluate content as answer to goal/ Expectation of adequacy of information	Monitor / review the content against the background of the objectives.<br>Statement that what is read and/or seen fits a goal or sub-goal.<br>Judging the usefulness of information (e.g. reading a certain text, watching a certain video, ...)<br>Statement that mine does not find a piece of information that one is looking for<br>Expectation that a certain form of representation is appropriate to achieve a goal	"I read the info, but it's not specific enough for what I'm looking for."<br>"... the info that seems important to me ..."<br>"... until I got to a part that was relevant to me."<br>"Texts that I felt were not relevant, I ignored."<br>Learner reads a text - "So, I think that's the answer to this question."<br>Structures of the heart ... here we are!"<br>"The video may give me the info I need to answer the question."	Merged with Evaluate content as answer to goal/ Expectation of adequacy of information
10) Monitor progress toward goals	Assessment of the extent to which set goals have been pursued	"Those were our goals, we achieved them."<br>"Then I checked again to make sure I had worked through all the points that were mentioned in the task."<br>"I finished the task."	
11) Monitor/ regulate use of strategies	Participants comment on how viable a strategy was/would have been had it been used or adjust their strategy as a result of monitoring<br>→ Also focus/ exert	"Yes, drawing it really helped me understand how the blood flows through our heart!"<br>"I guess I should have read the assignment sheet more carefully at the beginning."	Extended by Regulate use of strategies

Code	Description	Examples	Changes to G&A
**MACRO-LEVEL PROCESS: STRATEGY USE**			
13) Selecting a new informational source/ Read new text/ Reading, Re-Reading	Selecting and using various cognitive strategies for memory, learning, reasoning, problem solving, and thinking.<br>May include selecting a new representation, matching multiple representations, etc.<br>Selecting a new text that is different from the one the learner has just read.<br>Does not matter if learner is switching between multiple sheets. It does not have to be switching from book to page or vice versa<br>Reading a text<br>Looking at something<br>Read something in the environment again	[The learner learns about heart valves] and then moves on to watching the video to see their location.<br>"Okay, now on to the lungs." "I read through the text on XY."<br>"I'll read that again."<br>"To do that, I'm going to look again at the part about Paul and read it a little more carefully."<br>"The materials about Paul specifically I went through again in more detail."	Merged with Read new text, Reading, Re-reading
14) Coor-dinating informational sources	Coordinate different forms of representation (e.g. drawings and notes).<br>Also, when e.g. comparing own notes with other content or the content of different text sources	"I will bring this text together with the diagram."	
16) Review notes	Review notes	"I went back over all the points to be addressed in the conversation."	
17) Memo-rization	Trying to memorize texts, drawings, etc.	"I will try to memorize this picture."	
18) Free search	Searching the environment (the stack of papers) - without a specific plan or concrete goal.<br>→ together with (1 - Orientation/ Planning)	"I go to the beginning of the page / home page to see what's there."<br>"I tried to get a rough idea of the materials."	
19) Goal-directed search	Search the materials - according to a specific plan or concrete goal.	Learner types "blood circulation" into the search function<br>"...trying to still find content for the items for which I had not yet written anything or very little down."	
20) Summa-rization	Summarize what has been read, heard, or inspected in the environment	"This says white blood cells are involved in the destruction of foreign bodies."	

Code	Description	Examples	Changes to G&A
21a) Taking notes	Copy text passages from the environment/ note down key points	"I'm going to write this under heart."<br>"... wrote down the most important points as key words."<br>"... write down the criteria that apply to Paul."	
21b) High-lighting text passages	Mark / highlight places in the text	"... marked in the text."	Code added
22) Draw	Make tables, drawings or diagrams to better understand	"I try to mimic/draw the diagram as best I can."	
24) Inferences/ Hypo-thesizing	Draw conclusions - based on info read, heard, or seen in the environment<br>Ask questions that go beyond what has been read, heard, or seen	Lerner sees the image of the heart and remarks "So, the blood flows from here, to there, and on to here, ..."<br>"I wonder why only smooth walls in the vessels prevent blood clots from forming."	Merged with Hypothesizing
26) Knowledge elaboration	Link what they have read, heard and seen with existing knowledge<br>Thinking up examples<br>Imagine situations<br>→ If necessary, code with 3 - Prior knowledge activation	Lerner inspects an image of the main valves of the heart, noting "So this is how the circulatory and pulmonary systems work together."	
27) Mnemonic	Build mnemonic bridges to remember info (visualized, verbalized)	„Arteries – A for away."	
28) Find location in environ-ment	Statement where something was read in the environment.<br>Here especially when it comes to the stack of paper (that is our environment).	"That was where we were."<br>"The assignment sheet wasn't at the top of the pile, it was somewhere in between."	
29) Skip	Subject moves on to the next target before the previous target has been reached<br>Not when materials are skipped	"... can move on to the next..."	
**MACRO-LEVEL PROCESS: TASK DIFFICULTY AND DEMANDS**			
30) Time and effort planning	Attempts to control behavior.<br>→ Can also be coded together with 1 - Orientation/ Planning.<br>→ Do not code extra 11 - Monitor/ regulate use of strategies	"I'll skip this section. 45 minutes is too short to dive into all the details."	

Code	Description	Examples	Changes to G&A
31) Help seeking behavior	Seek help regarding appropriateness of answers or instruction.<br>Also when VP writes, for example, that she will look up terms she does not know.<br>Thus, help-seeking behavior does not necessarily have to refer to the experimenter, but can also include a search on the Internet or similar.	"Do you want me to give a more detailed answer?"	
32) Task difficulty	Learner expresses/demonstrates one of the following behaviors:<br>1) Task is difficult or easy<br>2) Questions are easy or difficult<br>3) Using the hypermedia environment is harder than reading a book	"This is harder than reading a book."	
33) Control of context/ Sorting, organising, grouping/ Cleaning space	Attempts to improve reading and seeing info.<br>Refers to materials, not notes	Learner sorts materials, forms various piles of paper Piles of paper<br>Learner tries to make space on his desk	Extended by Sorting, organizing, grouping und Cleaning space
**MACRO-LEVEL PROCESS: INTEREST**			
35a) Interest statement positive	Learner shows interest in the task or the content of the task; the task processing gives him/her pleasure	"Interesting."<br>"This stuff is interesting."	Extended by division into positive and negative
35b) Interest statement negative	Learner is unmotivated, bored with the task, ...		Extended by division into positive and negative
