# Supplementary material for: Misconceptions and Lack of Knowledge of Self-Regulation of Learning Hinder Students’ Use of Self-Regulation Strategies and Their Achievement: How This Can Be Changed by a Model-Based Instructional Video
Source: Behav Sci (Basel). 2026 Apr 20;16(4):612. doi: 10.3390/bs16040612 (PMC13113156; doi:10.3390/bs16040612)
Supplement: Supplementary file 1 [file behavsci-16-00612-s001.zip › Supplementary Materials S7.pdf]

Supplemental Material S7

Supplemental Material S7 shows the adaptation of the LIST to assess participants' (1) self-efficacy beliefs about using SRL strategies and (2) utility beliefs about SRL strategies instead of their use of SRL strategies.

Table S5

Adaptation of the LIST Questionnaire

	**Self-efficacy beliefs**	**Utility beliefs**
	I am able to…	I find it useful to…
Scale label	(1) cannot do it at all<br>(2) rather cannot do it<br>(3) can do it to some extent<br>(4) can do it rather well<br>(5) can do it certainly	(1) strongly disagree<br>(2) slightly disagree<br>(3) neither nor<br>(4) rather agree<br>(5) strongly agree
1	formulate a few questions before working on a text to give myself a goal.	
2	go through a particular passage again slowly if it seems confusing and unclear to me.	
3	set specific times at which I then study.	
4	think about the order in which I work through the material beforehand.	
5	recap the most important content without consulting my documents to identify gaps in my knowledge.	
6	think carefully beforehand about which parts of a particular subject area I need to learn and which I don't.	
7	make the manner in which I deal with the material dependent on the characteristics of the subject area.	
8	if I don't understand everything while reading a text, try to note down the gaps and then go through the text again.	
9	set a specific time period before each learning phase.	
10	at first skim the material (text or notes) to gain an impression of its content.	
11	work on additional tasks to determine whether I have really understood the material.	
12	adapt my learning technique to higher demands (e.g. by reading more slowly) if I encounter a difficult text.	
13	determine in advance how far I want to get with working through the material.	
14	make my learning approach dependent on the type and difficulty of the expected exam.	
15	set a schedule for the hours I spend studying each day.	
16	think about how I can proceed most effectively before learning a subject area.	
17	ask myself questions about the material to make sure I have understood everything.	
18	explain certain parts of the subject matter to a fellow student to check my own understanding.	
19	find out which subject areas I have not yet understood sufficiently.	
20	stick to a certain schedule when learning.	
