# Supplementary material for: Misconceptions and Lack of Knowledge of Self-Regulation of Learning Hinder Students’ Use of Self-Regulation Strategies and Their Achievement: How This Can Be Changed by a Model-Based Instructional Video
Source: Behav Sci (Basel). 2026 Apr 20;16(4):612. doi: 10.3390/bs16040612 (PMC13113156; doi:10.3390/bs16040612)
Supplement: Supplementary file 1 [file behavsci-16-00612-s001.zip › Supplementary Materials S8.pdf]

Supplemental Material S8

Supplemental Material S8 shows the results of repeated measures ANOVAs (if the normality assumption was not violated) and of Man-Whitney-*U*-tests (if the normality assumption was violated).

Table S6A

Results of Repeated Measures ANOVAs Comparing the Mastery with the Control Condition from Pre- to Post-Test

Variable	Effect	DFn	DFd	F	*p*
Self-efficacy beliefs	Group	1	106	0.04	.84
	Point of time	1	106	0.12	.73
	Group * Point of time	1	106	0.47	.50
Use of SRL strategies (LIST)	Group	1	106	1.07	.30
	Point of time	1	106	1.50	.22
	Group * Point of time	1	106	0.36	.55

Table S6B

Results of Man-Whitney-U-Tests Comparing the Mastery with the Control Condition from Pre- to Post-Test

Variable	W	*p*
Utility beliefs	1490	.90
Consistent beliefs	1328	.40
Inconsistent beliefs	1550	.70
Knowledge	1386	.90
Performance	1425	NA
Strategy use (reflection protocol)	1694	.20

Table S7A

Results of Repeated Measures ANOVAs Comparing the Intervention with the Control Condition from Pre- to Post-Test

Variable	Effect	DFn	DFd	F	P
Self-efficacy beliefs	Group	1	155	1.25	.27
	Point of time	1	155	0.24	.62
	Group * Point of time	1	155	1.01	.32
Utility beliefs	Group	1	155	1.96	.16
	Point of time	1	155	3.72	.06
	Group * Point of time	1	155	1.37	.24
Use of SRL strategies (retrospective reflection)	Group	1	154	0.02	.89
	Point of time	1	154	10.27	.002*
	Group * Point of time	1	154	0.07	.80

Table S7B

Results of Man-Whitney-U-Tests Comparing the Intervention with the Control Condition from Pre- to Post-Test

Variable	W	*p*
Strategy use (LIST)	2234	.10
Consistent beliefs	2442	.80
Inconsistent beliefs	3084	.02
Knowledge	1750	.002
Performance	2514	NA

Table S8A

Results of Repeated Measures ANOVAs Comparing the Intervention with the Control Condition from Post- to Follow-Up-Test

Variable	Effect	DFn	DFd	F	*p*
Self-efficacy beliefs	Group	2	136	1.98	.14
	Point of time	1	136	2.18	.14
	Group * Point of time	2	136	0.05	.95
	Group * Point of time	2	137	1.67	.19
Use of SRL strategies (LIST)	Group	2	136	1.02	.14
	Point of time	1	136	1.89	.17
	Group * Point of time	2	136	0.81	.45

Table S8B

Results of Man-Whitney-U-Tests Comparing the Intervention with the Control Condition from Post- to Follow-Up-Test

Variable	W	*p*
Utility beliefs	2090	.90
Consistent beliefs	1740	.30
Inconsistent beliefs	1894	.70
Knowledge	2396	.20
